# Roles of matricellular CCN2 deposited by osteocytes in osteoclastogenesis and osteoblast differentiation

**DOI:** 10.1038/s41598-019-47285-3

**Published:** 2019-07-29

**Authors:** Takashi Nishida, Satoshi Kubota, Hideki Yokoi, Masashi Mukoyama, Masaharu Takigawa

**Affiliations:** 1 Department of Biochemistry and Molecular Dentistry, Okayama University Graduate School of Medicine, Dentistry and Pharmaceutical Sciences, Okayama, Japan; 2 Advanced Research Center for Oral and Craniofacial Sciences, Okayama University Dental School, Okayama, Japan; 3 Department of Nephrology, Graduate School of Medicine, Kyoto University, Kyoto, Japan; 4 Department of Nephrology, Kumamoto University Graduate School of Medical Science, Kumamoto, Japan

**Keywords:** Targeted bone remodelling, Bone development

## Abstract

In this study, we investigated the effect of CCN2 (cellular communication network factor 2), previously termed connective tissue growth factor, deposited in bone matrix on osteoclastogenesis and osteoblast differentiation. To mimic the bone matrix environment, osteocytic MLO-Y4 cells had been embedded in collagen-gel with recombinant CCN2 (rCCN2), and mouse macrophage-like RAW264.7 cells were inoculated on the gel and treated with receptor activator of NF-κB ligand (RANKL). NFATc1 and cathepsin K (CTSK) productions were more increased in the combination of RAW264.7 and MLO-Y4 cells treated with rCCN2 than the combination without rCCN2. Next, we isolated an osteocyte-enriched population of cells and osteoclast progenitor cells from wild type and tamoxifen-inducible *Ccn2*-deficient (KO) mice and performed similar analysis. NFATc1 and CTSK productions were decreased in the KO osteocyte-enriched population at 6 months after the tamoxifen injection, regardless of the origin of the osteoclast progenitor cells. Interestingly, CTSK production was rather increased in KO osteocytes at 1 year after the injection. Finally, the combination of osteoblastic MC3T3-E1 and MLO-Y4 cells in rCCN2-containing bone matrix revealed the up-regulation of osteoblastic marker genes. These findings suggest that CCN2 supplied by osteocytes regulates both osteoclastogenesis and osteoblast differentiation.

## Introduction

Bone is continuously rebuilt by the cooperative action of bone-resorbing osteoclasts and bone-forming osteoblasts^1^. This process, termed “bone remodeling,” is thought to be important to maintain both the architecture and strength of bone tissues^1,2^. Recently, an *in vitro* culture system for osteocytes was established; and, as a result, these cells were found to have multiple functions, such as mechanosensing in response to mechanical loadings and regulation of bone remodeling^3^. Osteocytes are terminally differentiated osteoblasts and are the most abundant cells in bone tissues^3^. It is also well-known fact that osteocytes have many cell processes extending through canaliculi to form numerous connected communication networks between themselves and cells on the bone surface^3,4^. Therefore, it is believed that many extracellular communications with neighboring osteocytes, osteoblasts, and osteoclasts play an important role for osteocytes by acting as mechanosensory and regulatory cells of bone remodeling^3–5^. Osteocytes are not necessarily all in the same stage of differentiation^3,6^. Osteocytes localized around the bone surface extend many cell processes, are young, and capable of bone-forming activity^3,6^. In fact, it was reported that receptor activator of NF-κB ligand (RANKL), which has a critical role in osteoclastogenesis, is expressed in young osteocytes^7^, as observed in osteoblasts and chondrocytes^8,9^. On the other hand, older osteocytes, which are embedded deep in the bone matrix, are characterized by few cytoplasmic organelles and thin cell bodies, and do not express RANKL and bone formation markers^6,7^. These findings suggest that the morphology and functions of osteocytes change in a time-dependent manner.

Osteocytes function as mechanosensors within bone^3^. Mechanical stress loaded onto bone is sensed by osteocytes via their cell projections that are spread throughout the entire bone tissues, and physical stimulation is translated into biochemical signals^3,4^. These signals are believed to regulate the actions of osteoblasts and osteoclasts, thereby providing a mechanism to regulate bone formation and resorption according to the local microenvironment of the bone^3^. However, the biochemical signals coming from osteocytes are still unknown. We hypothesized that one of these biochemical signaling molecules could be CCN2 (cellular communication network factor 2), which was previously named connective tissue growth factor, because we earlier clarified that CCN2 was induced by mechanical stress and promotes the differentiation of osteoblasts as well as osteoclast formation^10–13^.

CCN2 is known as an extracellular matrix-associated (matricellular) protein. It is characterized by the presence of 4 distinct modules, i.e., insulin-like growth factor-binding protein (IGFBP), von Willebrand factor type C (VWC), thrombospondin type 1 repeat (TSP1), and a carboxyl terminal cystine knot (CT), which modules follow an N-terminal secretory signal^14–16^. It was reported that each module of CCN2 interacts with membrane proteins such as integrins as well as with many growth factors involved in bone remodeling^14–16^, thus suggesting that CCN2 may control a network of growth factors and membrane proteins during bone remodeling. We also reported that CCN2 promotes osteoblast differentiation^12^ and osteoclast formation^13^ *in vitro*. In addition, the gene expression and protein production of CCN2 were observed in osteocytes of alveolar bone during experimental tooth movement in mice^17^. Taken together, these findings suggest that, when mechanical stress is applied to osteocytes in bone tissues, CCN2 produced by osteocytes may promote the differentiation of neighboring osteoblasts as well as the formation of osteoclasts, via their cell projection network.

Conventional *Ccn2*-deficient mice die soon after birth due to respiratory distress caused by malformation of the rib cage as a result of severe skeletal abnormalities^18^. In fact, primary chondrocytes derived from the rib cage and osteoblasts from the calvariae of *Ccn2*-knockout mice at E18.5 exhibit impaired DNA synthesis and faulty extracellular matrix production^19^. Tartrate-resistant acid phosphatase (TRAP)-positive giant cells along with the bone trabeculae are also decreased in number in *Ccn2* mutants^13^. In addition, neonatal *Ccn2*-deficient mice show deformed long bones^18^. These findings indicate that not only osteoblastogenesis and osteoclastogenesis, but also bone remodeling, which is the balance of bone formation and bone resorption may be impaired in *Ccn2*-deficient mice. Therefore, it is possible that CCN2 and osteocytes embedded in bone matrix may together regulate bone remodeling. To clarify this point, *Ccn2*-deficient osteocytes are needed. However, osteocytes cannot be isolated from conventional *Ccn2*-deficeint mice because of the neonatal death of these mice^18^. Therefore, we employed tamoxifen-inducible conditional *Ccn2*-deficent mice^20,21^. These mice were born at expected Mendelian ratios, displayed no obvious skeletal abnormalities at birth, and grew normally to adulthood, with their weight being the same as that of wild type mice^20,21^. Here, the aim of our study is to clarify the effect of CCN2 from osteocytes on bone remodeling. We investigated the roles of CCN2 in osteoclastogenesis and osteoblast differentiation by using a 3-D culture system of a murine osteocytic cell line, MLO-Y4 cells^22^, and an osteocyte-enriched cell population isolated from tamoxifen-inducible conditional *Ccn2*-deficient mice. Here we found that osteocyte-derived CCN2 modified both osteoblast differentiation and osteoclast formation, indicating CCN2 to be an essential factor of bone remodeling.

## Results

### Down-regulation of *Opg* in MLO-Y4 cells embedded into collagen gel containing recombinant CCN2 protein (rCCN2)

**Figure 1:**
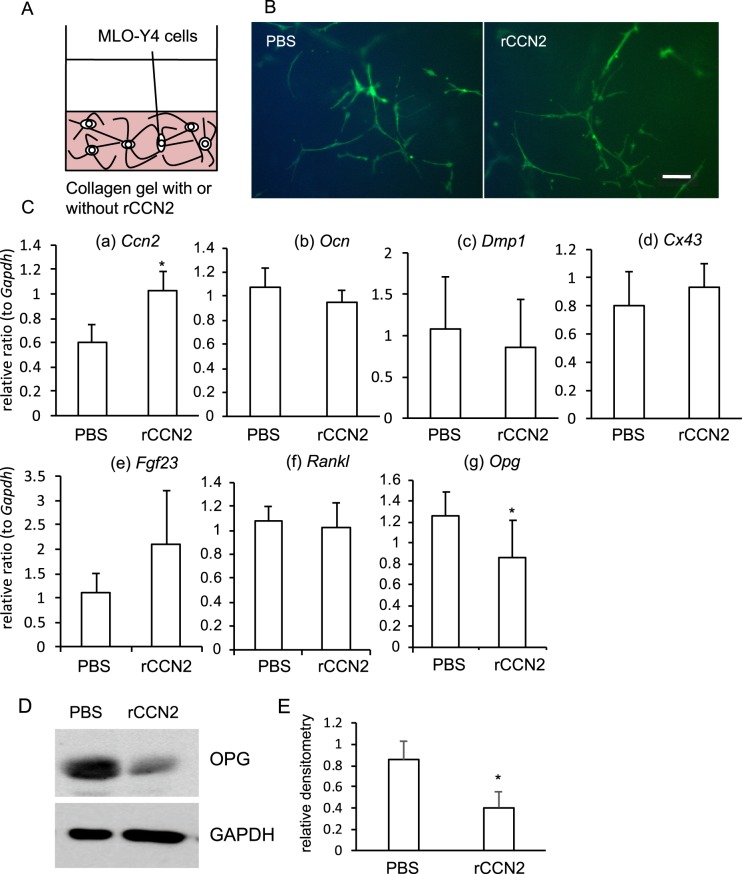
Gene expression of osteocyte markers and OPG production in MLO-Y4 cells embedded into collagen gel including rCCN2. (**A**) Illustration of the 3-D culture system of MLO-Y4 cells. (**B**) Fluorescence staining to visualize MLO-Y4 cells embedded into collagen gel with or without rCCN2. MLO-Y4 cells were embedded into collagen gel containing rCCN2 (300 ng/ml) and cultured for 2 days. Then, fluorescence staining was performed by using fluorescein phalloidin. MLO-Y4 cells with cell projections were detected, but CCN2 in the gel showed no effect. PBS was added to the gel as the vehicle. The bar represents 100 µm. (**C**) After MLO-Y4 cells had been embedded into collagen gel with rCCN2, they were cultured for 2 days. Total RNA was isolated and real-time RT-PCR analysis was performed by using specific primers. The amounts of osteocyte marker genes were normalized to that amount of *Gapdh* mRNA. Data show the value from independent samples of n = 6, and the graph presents the mean and standard deviation. The asterisk indicates a significant difference from PBS treatment (*p* < 0.05). (**D**) Western blot analysis was performed by using anti-OPG and GAPDH antibodies in MLO-Y4 cells embedded into the gel with or without rCCN2. (**E**) The amount of OPG was determined densitometrically, and these amounts were normalized to the amounts of GAPDH. Graph shows the relative density of OPG with (n = 3) or without rCCN2 (n = 3). Bars represent mean and standard deviation. The data were analyzed by Student *t*-test, and *p* < 0.05 (*) was considered significant.

CCN2 is known to be present in bone tissues^23^. In order to mimic the bone microenvironment, we established a three-dimensional (3-D) culture system, in which MLO-Y4 cells are embedded into collagen gel with rCCN2 (300 ng/ml; Fig. 1A). Using this system, we firstly investigated whether or not the morphology of MLO-Y4 cells would be affected by rCCN2 in the collagen gel. After cultivation for 2 days, the cells were fixed and fluorescent-phalloidin staining was performed. As a result, the morphology of MLO-Y4 cells embedded in the collagen gel showed no difference by the treatment with either PBS or rCCN2 (Fig. 1B). Next, we examined the gene expression levels of osteocyte markers, such as *Osteocalcin* (*Ocn*), *Dentin matrix protein 1* (*Dmp1*), *Connexin 43* (*Cx43*) and *Fibroblast growth factor 23* (*Fgf23*). As shown in Fig. 1C, although the gene expression of *Ccn2* was up-regulated in MLO-Y4 cells embedded in the collagen gel with rCCN2, there was no effect on the gene expression of osteocytic markers (Fig. 1C). However, we showed that the gene expression and protein production of osteoprotegerin (OPG), which is a decoy receptor of RANK, was significantly decreased by rCCN2 embedded in the gel, although RANKL expression showed no difference (Fig. 1C–E). Since we had earlier reported that CCN2 plays an important role in bone formation^12^ and bone resorption^13^, this finding suggests that CCN2 affected osteoclastogenesis via osteocytes but that osteocytic marker gene expressions were not involved.

### MLO-Y4 cells promoted osteoclast formation from RAW264.7 cells in 3-D culture with rCCN2

**Figure 2:**
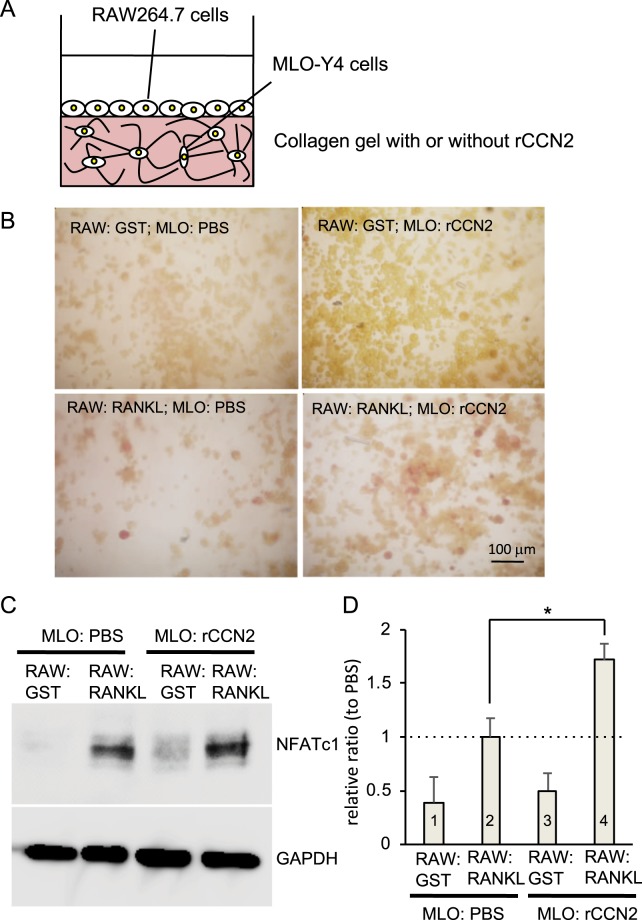
Effect of MLO-Y4 cells embedded into the gel with rCCN2 on osteoclastogenesis of RAW264.7 in the presence of GST-RANKL. (**A**) Illustration of co-culture system of collagen gel-embedded MLO-Y4 cells and RANKL-treated RAW264.7 cells. (**B**) MLO-Y4 cells were embedded into collagen gel containing rCCN2 (300 ng/ml) at a density of 2 × 10^5^/well in 12-well plate. After having been cultivated for 1 day, RAW264.7 cells were inoculated at a density of 4 × 10^4^/well onto the gel and treated with GST-RANKL or GST for 2 days. Then, TRAP staining was performed. The bar represents 100 µm. (**C**) Under the same conditions indicated in “B,” cell lysate was prepared and collected; and Western blot analysis was performed by using anti-NFATc1 and GAPDH antibodies. The results shown are representative of 3 individual experiments. (**D**) The amount of NFATc1 was determined densitometrically and normalized to the amount of GAPDH. Data show the mean and standard deviation of 3 individual experiments. The ordinate indicates the fold change relative to column 2 (ratio = 1.0; dotted line). These data were analyzed by performing Bonferroni’s test, and *p* < 0.05 (*) was considered significant.

Next, to investigate whether or not MLO-Y4 cells with rCCN2 would promote osteoclastogenesis, we constructed a 3-D culture system of MLO-Y4 cells in a collagen gel containing rCCN2 and thereafter inoculated RAW264.7 cells onto the collagen gel. Then, the RAW264.7 cells were treated with Glutathione S-transferase (GST)-fused RANKL (GST-RANKL; Fig. 2A). As shown in Fig. 2B, TRAP-positive giant cells were detected in cultures treated with GST-RANKL, and these cells were further increased in number when rCCN2 was present in the gel. On the other hand, as a control experiment, treatment with GST protein slightly yielded TRAP-positive cells on the gel with rCCN2 and few TRAP-positive cells were found on the gel with PBS (Fig. 2B). To support these results, we investigated the protein level of nuclear factor of activated T cells (NFATc1), which is a critical transcriptional factor in osteoclast differentiation, in RAW264.7 cells cultured on the gel containing MLO-Y4 cells with or without rCCN2. As shown in Fig. 2C,D, the level of NFATc1 was significantly increased in RANKL-stimulated RAW264.7 cells on the gel including MLO-Y4 cells with rCCN2, compared with that on the gel with PBS. Interestingly, NFATc1 was slightly detected in RAW264.7 cells on the gel including MLO-Y4 cells and rCCN2 even by the treatment with GST, although densitometric analysis of the band showed no significant difference (Fig. 2D). This finding indicates that CCN2 in the gel may have stimulated osteoclast formation by RAW264.7 cells directly. Since CCN2 is known to be regulated by Sox9 that plays a critical role in chondrogenesis^24^, we transfected MLO-Y4 cells with a Sox9 expression vector containing 3 × HA tag by using electroporation and examined whether CCN2 was regulated by Sox9, or not (Fig. S1A). After transfection, total RNA was isolated and real-time PCR analysis was performed. As shown in Fig. S1B, *Ccn2* expression showed no change in MLO-Y4 cells by the transfection of the Sox9 expression vector.

### Osteoclastogenesis from RAW264.7 cells seeded on the gel containing MLO-Y4 cells transfected with a *Ccn2* expression vector

**Figure 3:**
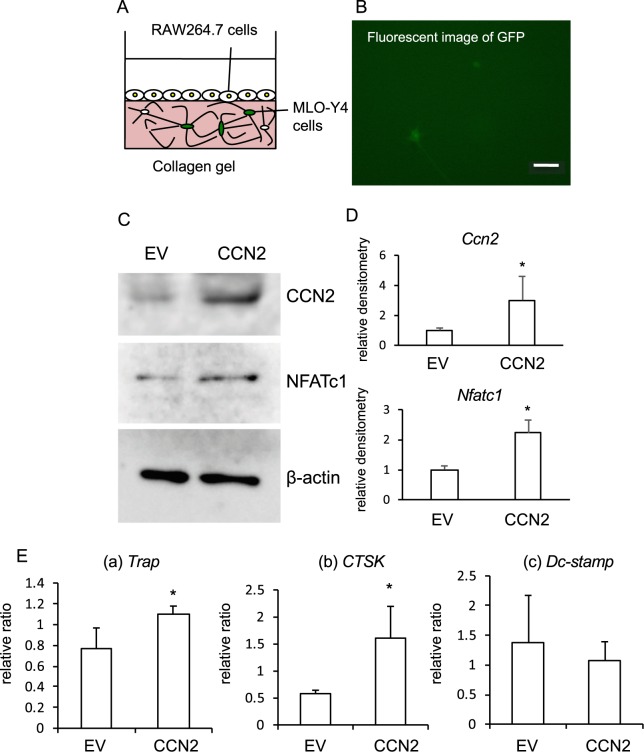
Effect of the gel-embedded MLO-Y4 cells transfected with *Ccn2* and *GFP* expression vectors on osteoclastogenesis of RAW264.7 cells in the presence of GST-RANKL. (**A**) Illustration of co-culture system of *Ccn2* overexpressed MLO-Y4 cells and RAW264.7 cells. (**B**) MLO-Y4 cells were transfected with both *Ccn2* and GFP expression vectors by using electroporation, and embedded into collagen gel at a density of 6 × 10^5^ cells/well in 12-well plate. After the cells had been cultivated for 3 days, GFP was detected by using fluorescence microscopy. The bar represents 100 µm. (**C**) After having been cultivated for 3 days, RAW264. 7 cells were inoculated onto the gel and treated with GST-RANKL for 2 days. Cell lysates were collected, and Western blot analysis was performed by using anti-CCN2, NFATc1 and β-actin antibodies. (**D**) The amounts of CCN2 (n = 5) and NFATc1 (n = 3) were determined densitometrically and were normalized to that of β-actin. The graph indicates density relative to that with empty vector (EV) (ratio = 1.0) and was analyzed by Student’s *t-*test; *p* < 0.05 (*) was considered significant. (**E**) Total RNA was isolated, and real-time RT-PCR analysis was performed by using specific primers for *Trap*, *Ctsk*, *Dc-stamp* and *Gapdh*. The amounts of these transcripts were normalized to that amount of *Gapdh* mRNA. Data show the value from independent samples of n = 4, and the graph presents the mean and standard deviation. The asterisk indicates a significant difference from the cells transfected with EV (*p* < 0.05).

To confirm that nascent CCN2 produced by osteocytes promotes osteoclast formation of RAW264.7 cells, we performed transient transfection of MLO-Y4 cells with *Ccn2* and *green fluorescent protein* (*Gfp)* expression plasmids by using electroporation, and these cells were embedded in a collagen gel. Then, RAW264.7 cells were inoculated onto the gel and treated with GST-RANKL (Fig. 3A). As shown in Fig. 3B, fluorescent signals of GFP were detected in the MLO-Y4 cells, suggesting that *Ccn2* expression plasmid together with pmax GFP vector had successfully entered the MLO-Y4 cells. To support these data, we analyzed the CCN2 protein and found that production of CCN2 was further increased in the cells with *Ccn2* expression vector than in those with the empty vector. Under these conditions, NFATc1 production was also significantly increased (Fig. 3C,D). In addition, the expression levels of osteoclast differentiation marker genes, *Trap* and *Cathepsin K* (*Ctsk*), were significantly up-regulated in RAW264.7 cells seeded on the gel containing MLO-Y4 cells overexpressing CCN2; but *Dendritic cell-specific transmembrane protein* (*Dc-stamp*) expression was unaffected (Fig. 3E). These results suggest that CCN2 produced by MLO-Y4 cells promoted the osteoclast formation from RAW264.7 cells.

### Isolation of osteocyte-enriched cell population from the femur of tamoxifen-inducible *Ccn2*- deficient mice

**Figure 4:**
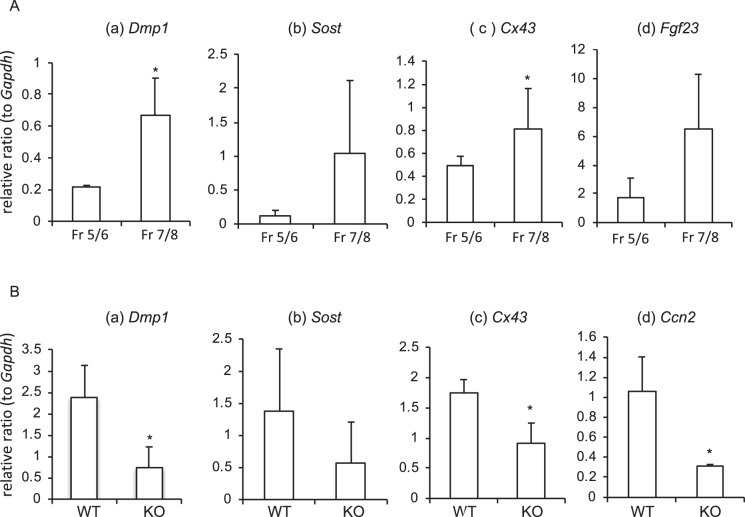
Gene expression of osteocytic markers in cell fractions enzymatically isolated from mouse long bone, and in the osteocyte-enriched cell population from wild type and tamoxifen-inducible *Ccn2*-deficient mice. (**A**) After bone marrow had been flashed out, femurs were cut into small pieces and digested by 0.2% collagenase for 20 min. The supernatant was saved as Fr1, and the bone pieces were treated with 5 mM EDTA for 20 min. The supernatant was saved as Fr2. This was repeated and the cells of Fr5/6 and Fr7/8 were collected. After Fr5/6 and Fr7/8 had been collected and placed in 2-D cultures for a few days, these cells were embedded into collagen gel and cultured for 5 days. Total RNA was isolated, and real-time RT-PCR analysis was performed by using specific primers. The amounts of osteocyte marker transcripts were normalized to that of *Gapdh* mRNA. Data show the value from independent samples of n = 6, and graph presents the mean and standard deviation. The asterisk indicates a significant difference from the Fr5/6 sample (*p* < 0.05). (**B**) Femurs were collected from tamoxifen-inducible *Ccn2*-deficient and wild type mice 1 month after injection with tamoxifen. As described in “A,” osteocytes in osteocyte-enriched cell population (Fr7/8) were isolated. After 2-D culture for a few days, these cells were embedded into collagen gel and cultured for 5 days. Total RNA was isolated and real-time RT-PCR analysis was performed by using specific primers. Data show the value from independent samples of n = 6, and the graph presents the mean and standard deviation. The asterisk indicates a significant difference from WT osteocyte-enriched cells (*p* < 0.05).

Next, to investigate the effect of CCN2 loss of function on osteocyte functions, we firstly digested murine femur enzymatically according to a previous report^25^. Briefly, we isolated osteocyte-enriched fractions from bone marrow-removed long bone by serial digestion with collagenase and decalcification by ethylenediaminetetraacetic acid (EDTA). During fractionation, we collected and pooled the late fractions (Fr5-8). As shown in Fig. 4A, gene expression of osteocyte-markers, such as *Dmp1* and *Cx43*, was up-regulated significantly in the cells from Fr7/8 compared with that for the cells from Fr5/6; and *Sost* and *Fgf23* gene expression levels tended to be increased in Fr7/8 as well. Based on these results, we used the cells from Fr7/8 as the osteocyte-enriched population to carry out all subsequent studies. Of note, expression of *Ccn2* in those cells was confirmed for the first time (Fig. 4B). Next, to investigate the effect of *Ccn2* deficiency on osteocyte functions, we used osteocytes from tamoxifen-inducible *Ccn2*-deficient mice. Tamoxifen-inducible *Ccn2*-deficient mice were generated by crossing *Ccn2*^flox/flox^ mice with CAG-Cre^Esr1^ mice as described in “Methods”. PCR analysis using genomic DNA showed efficient deletion of *Ccn2* in the tail of CAG-Cre^Esr1^;*Ccn2*^flox/flox^ mice 1 month after injection with tamoxifen (Fig. S2A,B). Efficiency of *Ccn2* depletion in the osteocyte-enriched cell population of CAG-Cre^Esr1^;*Ccn2*^flox/flox^ mice was approximately 80% (Fig. 4B, d) and osteocytic marker genes, such as *Dmp1*, and *Cx43*, were significantly repressed (Fig. 4B, a and c). *Sost* gene expression was unaffected (Fig. 4B, b). Although CAG-Cre^Esr1^;*Ccn2*^flox/flox^ mice grew normally in appearance^20,21^ and exhibited no obvious skeletal abnormalities, these results suggest that osteocyte functions may have been impaired in these mice.

### Impaired osteoclastogenesis by *Ccn2*-deficient osteocyte-enriched population

**Figure 5:**
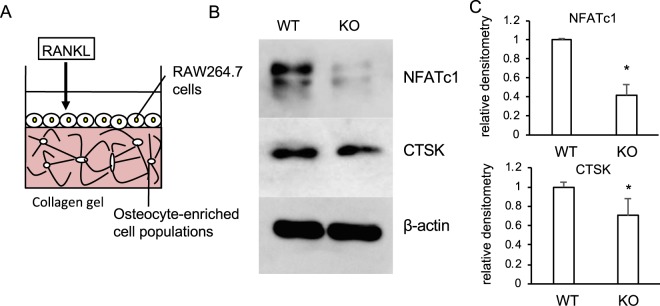
Effect of *Ccn2*-deficient osteocytes on osteoclastogenesis of GST-RANKL-treated RAW264.7 cells. (**A**) Illustration of co-culture system of collagen gel-embedded WT and KO osteocyte-enriched population of cells and RANKL-treated RAW264.7 cells. (**B**) WT and KO mice at 1 month of age were injected with tamoxifen, and after 1 month, femurs were collected. Then, osteocyte-enriched cell population was isolated from WT and KO mice and cultured for 2 days. Thereafter, WT and KO osteocyte-enriched populations were embedded into collagen gel at a density of 5 × 10^4^/well in 24-well plates. After having been cultivated for 2 days, RAW264.7 cells were inoculated onto the gel and treated with GST-RANKL for 2 days. Then, cell lysates were prepared; and Western blot analysis was performed by using anti-NFATc1, CTSK and β-actin antibodies. (**C**) The amounts of NFATc1 and CTSK were determined densitometrically and were normalized to the amount of β-actin. Graph shows the relative density of NFATc1 (upper) and CTSK (lower) in WT (n = 3) or KO cells (n = 3). In these graphs, the ordinate indicates the relative ratio with respect to the sample from WT cells (ratio = 1.0), and bars represent mean and standard deviation. The data were analyzed by Student *t*-test, and *p* < 0.05 (*) was considered significant.

**Figure 6:**
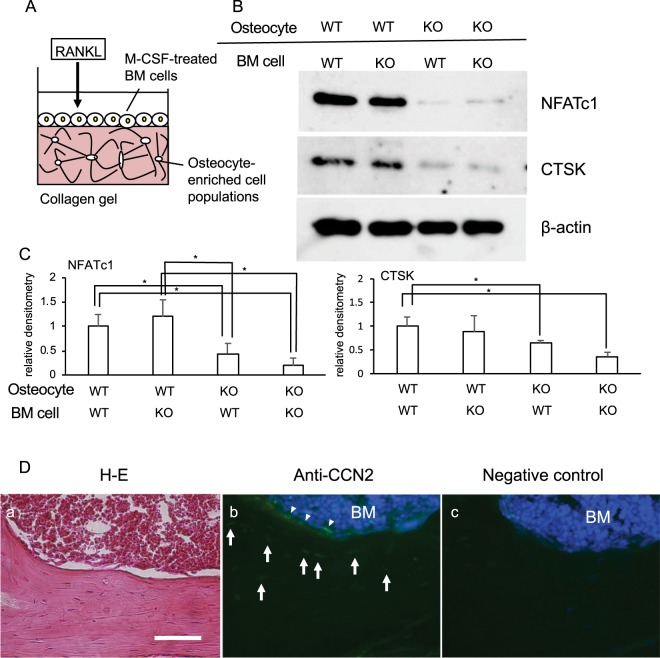
Effect of combination of osteocytes and M-CSF/RANKL-treated bone marrow (BM) cells from *Ccn2*-deficient mice at 7 months-old on osteoclastogenesis. (**A**) Illustration of co-culture system of collagen gel-embedded WT or KO osteocyte-enriched population and BM cells treated with M-CSF and RANKL. (**B**) WT and KO mice at 1 month-old were injected with tamoxifen; and after 6 months, femurs were collected. After the bone marrow had been removed, BM cells were isolated from it and cultured for 1 day. Floating cells were re-plated and treated with M-CSF (50 ng/ml) for 2 days. The osteocyte-enriched population was isolated from the remaining bone tissues and cultured for 3 days. Then, the WT or KO osteocyte-enriched cell population of cells at a density of 2.5 × 10^4^/well were embedded into collagen gel in 24-well plates; and the M-CSF-treated adherent BM cells (1.5 × 10^5^ cells/ml) were inoculated onto the gel and treated with RANKL (100 ng/ml) for 10 days, with media containing RANKL being refreshed every 3 days. Then, cell lysate was prepared; and Western blot analysis was performed by using anti-NFATc1, CTSK and β-actin antibodies. (**C**) The amounts of NFATc1 and CTSK were determined densitometrically and were normalized to that of β-actin. Left graph shows the relative density of NFATc1 in the combination of WT osteocytes with WT BM cells (WT/WT; n = 6), WT osteocytes with KO BM cells (WT/KO; n = 5), KO osteocytes with WT BM cells (KO/WT; n = 6) and KO osteocytes with KO BM cells (KO/KO; n = 3). Right graph shows the relative density of CTSK in WT/WT (n = 5), WT/KO (n = 4), KO/WT (n = 5) and KO/KO (n = 3). In these graphs, the ordinate indicates the relative ratio with respect to the data of WT/WT (ratio = 1.0), and bars represent mean and standard deviation. The data were analyzed by Bonferroni’s test, and *p* < 0.05 (*) was considered significant. (**D**) Immunoreactivity for CCN2 was detected in osteocytes (arrows) and osteoblasts (arrowheads) of wild type murine cortical bone (7 months-old; b). Serial section was stained with hematoxylin-eosin (H-E; a), or without primary antibody as a negative control (c). The scale bar represents 100 μm. BM represents bone marrow.

We next used the osteocyte-enriched population of cells from the femur of CAG-Cre^Esr1^;*Ccn2*^flox/flox^ mice and examined whether or not these cells would modify the osteoclastogenesis of RAW264.7 cells treated with GST-RANKL. As shown in Fig. 5A, the *Ccn2*-deficient osteocyte-enriched population of cells was embedded into collagen gel, and RAW264.7 cells were then seeded onto the gel. Cell projections showed no difference between control and *Ccn2*-deficient cells in the gel (Fig. S3A,B). However, both NFATc1 and CTSK production levels were decreased in RANKL-stimulated RAW264.7 cells on the gel containing the *Ccn2*-deficient osteocytes (Fig. 5B,C). These findings indicate that CCN2 produced by osteocytes modified osteoclastogenesis, but had no effect on the morphology of the cells in osteocyte-enriched population. Because we previously showed that CCN2 produced by osteoclast progenitor cells also plays an important role in osteoclastogenesis^13^, we next compared the production of NFATc1 and CTSK in the presence of CCN2 produced by the osteoclast progenitor cells or by osteocytes. As shown in Fig. 6A, we isolated bone marrow (BM) cells from the femur of *Ccn2*^flox/flox^ (wild-type; WT) or CAG-Cre^Esr1^;*Ccn2*^*f*lox/flox^ (KO) mice 6 months after injection with tamoxifen (7 months old), and inoculated BM cells stimulated by macrophage colony-stimulating factor (M-CSF) onto the collagen gel containing the osteocyte-enriched population of the cells isolated from the same mice. Cell projections in the osteocyte-enriched population from WT and KO cells in the collagen gel showed no difference (Fig. S3C,D). Under these conditions, we performed Western blot analysis using anti-NFATc1 and CTSK antibodies. As shown in Fig. 6B,C, the production levels of NFATc1 and CTSK in the osteocyte-enriched population of cells from WT mice were higher than those for KO mice, regardless of the source of BM cells. These findings suggest that CCN2 derived from osteocytes played a more important role in osteoclastogenesis than CCN2 from osteoclast progenitor cells. Finally, to examine if CCN2 is expressed in osteocytes *in vivo*, we performed immunofluorescence staining of WT mice at 7-months old by using an anti-CCN2 antibody. As shown in Fig. 6D, CCN2 was produced in osteocytes around bone surface (arrows) and osteoblasts on bone surface (arrowheads). However, CCN2 was not detected in cortical bone from aged mice (Fig. S4). These results confirm that CCN2 is expressed in young and activated osteocytes.

### Promotion of CTSK production by *Ccn2*-deficient osteocyte-enriched population of cells from aged mice

**Figure 7:**
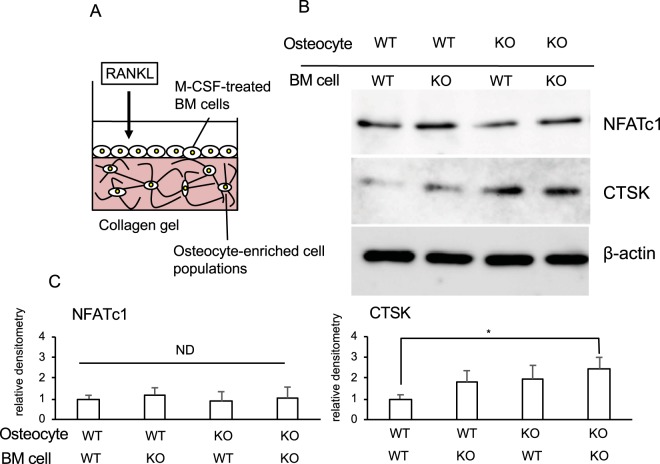
Effect of combination of osteocytes and M-CSF/RANKL-treated bone marrow (BM) cells from *Ccn2*-deficient aged mice on osteoclastogenesis. (**A**) Illustration of co-culture system of collagen gel-embedded WT or KO osteocyte-enriched cell population and M-CSF and RANKL-treated BMCs from aged mice. (**B**) At 1 year after tamoxifen injection, femurs of WT and KO mice were collected, and BM cells and remaining bone tissues were separated. BM cells were cultured for 1 day, and the floating cells were collected and treated with M-CSF (50 ng/ml) for 4 days. Osteocytes were isolated from remaining bone tissues and cultured for 5 days. WT or KO osteocytes were embedded into collagen gel in 24-well plate at a density of 2.0 × 10^4^/well, and the M-CSF-treated adherent BM cells (1.0 × 10^5^ cells/ml) were inoculated onto the gel and treated with GST-RANKL (100 ng/ml) for 10 days, with media containing GST-RANKL being refreshed every 3 days. Then, cell lysates were prepared, and Western blot analysis was performed by using anti-NFATc1, CTSK and β-actin antibodies. (**C**) The amounts of NFATc1 and CTSK were determined densitometrically and were normalized to the amount of β-actin. Graph shows the relative density of NFATc1 (left) and CTSK (right) in the combination of WT osteocytes with WT BM cells (WT/WT; n = 4), WT osteocytes with KO BM cells (WT/KO; n = 4), KO osteocytes with WT BM cells (KO/WT; n = 4) and KO osteocytes with KO BM cells (KO/KO; n = 4). In these graphs, the ordinate indicates the relative ratio with respect to the data of WT/WT (ratio = 1.0), and bars represent mean and standard deviation. The data were analyzed by Bonferroni’s test, and *p* < 0.05 (*) was considered significant. ND represents no difference.

To examine the effect of aging on the observed findings, we isolated osteocyte-enriched population of cells and BM cells from WT or KO mice 1 year after injection with tamoxifen (13 months-old), and constructed 3-D cultures of the osteocyte-enriched population of cells with the BM cells (Fig. 6A). As a result, the morphology was similar between aged WT and KO osteocytes (Fig. S3E,F). The decrease in protein levels of NFATc1 and CTSK in BMCs caused by young *Ccn2*-deficient osteocytes 6 months after the tamoxifen injection (Fig. 6B,C) was not observed anymore in osteocytes from KO mice 1 year after injection (Fig. 7B,C), suggesting a decrease in CCN2 production by osteocytes in aged mice. Interestingly, CTSK production was rather increased by the combination of the KO osteocyte-enriched population and KO BM cells, although NFATc1 production showed no difference between the two (Fig. 7B,C).

### MLO-Y4 cells promoted osteoblast differentiation in 3-D cultures with rCCN2

**Figure 8:**
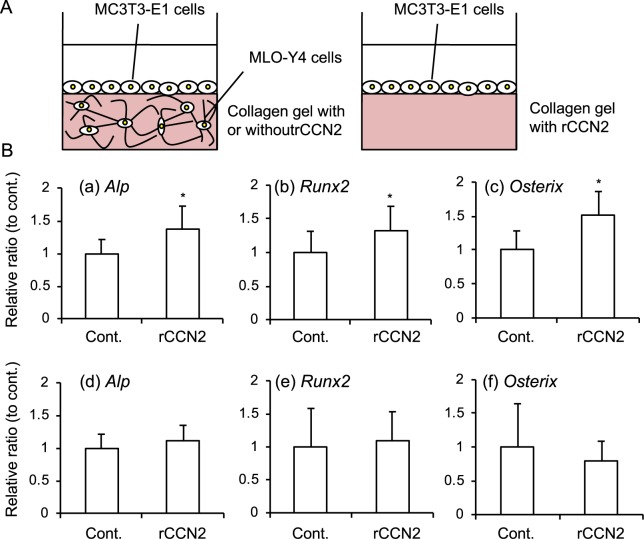
Effect of MLO-Y4 cells embedded into the gel with rCCN2 on osteoblast differentiation. (**A**) Illustration of co-culture system of MC3T3-E1 cells and MLO-Y4 cells (left panel). As a control, MC3T3-E1 cells were cultured on the gel including rCCN2 only (right panel). (**B**) MLO-Y4 cells were embedded into collagen gel with rCCN2 in 12-well plates, and cultured for 1 day. Then, MC3T3-E1 cells were inoculated onto the gel with or without MLO-Y4 cells at a density of 3 × 10^4^/well. After 24 h, total RNA was isolated; and real-time RT-PCR analysis was performed. The amounts of *Alp* (a and d), *Runx2* (b and e) and *Osterix* (c and f) were normalized to those of *Gapdh* mRNA. All data show the value from independent samples with (n = 12) or without (n = 12) rCCN2. The ordinate indicates the relative ratio with respect to the treatment with PBS (ratio = 1.0) in all graphs. The graphs (a–c) show the results from MC3T3-E1 cells on the gel with MLO-Y4 cells and graphs (d–f) show those for MC3T3-E1 cells only. Bars represent mean and standard deviation. The data were analyzed by Student’s *t*-test, and *p* < 0.05 (*) was considered significant.

Finally, we investigated the effect of MLO-Y4 cells embedded into collagen gel with rCCN2 on osteoblast differentiation from a murine osteoblastic cell line MC3T3-E1. As shown in Fig. 8A, we inoculated MC3T3-E1 cells on the gel containing MLO-Y4 cells with rCCN2 at the concentration of 300 ng/ml. In addition, to test the effect of MLO-Y4 cells embedded into the gel with rCCN2, we also inoculated MC3T3-E1 cells on the gel having no MLO-Y4 cells. After 24 h, total RNA was isolated; and real-time PCR analysis was performed by using specific primers for osteoblast differentiation markers, such as *Alkaline phosphatase* (*Alp*), *runt-related transcription factor 2* (*Runx2)* and *Osterix* (Table S1). The gene expressions of *Alp* (a), *Runx2* (b) and *Osterix* (c) were significantly up-regulated in the combination of MC3T3-E1 cells and MLO-Y4 cells embedded into collagen gel with rCCN2 (Fig. 8B; upper panel, a–c). On the other hand, these levels showed no difference in MC3T3-E1 cells seeded on the gel without MLO-Y4 cells (Fig. 8B; lower panel, d–f). These results indicate that CCN2 modified osteoblast differentiation and that the effect was more pronounced in the presence of osteocytes, suggesting that CCN2-stimulated osteocytes further promoted osteoblast differentiation.

## Discussion

We previously reported that CCN2 was strongly expressed in pre-hypertrophic chondrocytes of growth plate and that it promoted chondrocyte proliferation and differentiation^15,26^, osteoblast differentiation^12,15,23^, and osteoclast formation^13,15^. In addition, an *in vivo* study revealed that conventional *Ccn2-*deficient mice suffered neonatal death due to respiratory failure as a result of abnormal rib cage development during embryonic stages^18^. These findings suggest that CCN2 is a critical growth factor that dynamically regulates skeletogenic processes, such as endochondral ossification, during development^15^. On the other hand, the expression level of CCN2 is very low during the adult stage^27^; and its physiological roles at that time remained unknown. To clarify the roles of CCN2 during the adult stage, in this study we used CAG-Cre^Esr1^;*Ccn2*^flox/flox^ mice, in which the *Ccn2* gene in the whole body could be knocked down by injection with tamoxifen after birth^20,21^. The mice that received tamoxifen injection starting at 1 month of age displayed no obvious skeletal abnormalities by 6 months or 1 year after the injection with tamoxifen, although their weight was reduced compared with that of the control mice 1 year after the injection with tamoxifen (Fig. S2C). However, a preliminary histological analysis has shown that bone mass tended to be decreased in secondary ossification centers of CAG-Cre^Esr1^;*Ccn2*^flox/flox^ mice tibiae (Fig. S2D), suggesting that CCN2 is somehow involved in bone remodeling, although further investigation needs to be carried out to confirm these findings. Apart from such preliminary data, we suspect that CCN2 locally induced during tissue regeneration, such as during bone or cartilage wound healing, plays major roles in such tissue regeneration, based on previous results^28,29^. Furthermore, it is reported that the CCN2 gene expression level is increased in the trabecular bone of the human lumbar spine, which is always exposed to mechanical loadings, compared with that in trabecular bone of the human iliac crest, where no mechanical loading occurs^30^. Because there are common biological processes between bone remodeling and bone regeneration, these findings together suggest that CCN2 induced by mechanical loading has an important role in bone remodeling, even in adult tissues.

Because it is well-known fact that osteocytes sense mechanical stresses loaded onto bone tissues^3,4^, we considered that investigating the roles of CCN2 in bone tissue might lead to elucidation of the molecular mechanism of bone remodeling regulated by osteocytes. Since adequate bone remodeling is performed under the balance of bone formation and bone resorption^1,2^, we firstly investigated how CCN2 present with osteocytes affected osteoclast formation. We believe that our 3-D culture system of MLO-Y4 cells and osteocyte-enriched cell population in collagen gel is biologically similar to the environment of bone tissues *in vivo*, because these cells extend cell projections that connect the neighboring cells therein (Figs 1B and 3B)^5^. Therefore, 3-D cultures of MLO-Y4 cells in collagen gel containing rCCN2 were prepared; and RAW264.7 cells were inoculated onto it. As a result, MLO-Y4 cells embedded in the rCCN2 containing gel promoted osteoclastogenesis of RAW264.7 cells on the gel (Fig. 2C,D). It is reported that integrin α_v_β_3_ attaches between cell processes and the extracellular matrix (ECM)^31^. Since it is well-known that CCN2 interacts with integrin α_v_β_3_^14^, CCN2 may act through its binding to integrin α_v_β_3_ on MLO-Y4 cells embedded in collagen gel. Additionally, we showed that *Ccn2* gene was significantly up-regulated in MLO-Y4 cells by rCCN2 in the collagen gel (Fig. 1C; a). This result indicates that *Ccn2* in MLO-Y4 cells is regulated in an autocrine manner by the rCCN2 in the gel, probably through the integrin signaling. Moreover, to clarify the roles of CCN2 in osteocyte functions more directly, we isolated the osteocyte-enriched population of cells from tamoxifen-inducible *Ccn2*-deficient mice 6 months after injection with tamoxifen and prepared 3-D cultures in the collagen gel. BM cells, which were isolated from the bone marrow of the same mice, were treated with M-CSF. Then, M-CSF-treated BM cells were inoculated onto the gel and treated with RANKL. As shown in Fig. 6B,C, osteoclastogenesis was impaired in cultures of cells in the *Ccn2*-deficient osteocyte-enriched population, even if BMCs were from either WT or KO mice. These results suggest that CCN2 from osteocytes promoted osteoclastogenesis. It was reported that osteocytes regulate bone resorption via RANKL and OPG production^32,33^. As shown in Fig. 1C–E, when MLO-Y4 cells were stimulated by rCCN2, the gene expression and protein production of OPG were significantly decreased. Therefore, we suspected that OPG expression was increased by the deletion of CCN2 in osteocytes. However, when we investigated the gene expression levels of RANKL and OPG in the osteocyte-enriched population derived from WT and KO mice, these gene expression levels showed no difference between the 2 sources (Fig. S5). Taken together, these results suggest that the effect of osteocyte-produced CCN2 on osteoclast formation was not mediated via RANKL and OPG productions, but was directly conferred by CCN2.

The reductions in protein levels of NFATc1 and CTSK in BM cells caused by osteocytes derived from KO mice 6 months after injection with tamoxifen (Fig. 6B,C) were observed like in the case of osteocytes derived from KO mice 1 month after the injection (Fig. 5B,C), but the reduction was not observed in osteocytes from KO mice 1 year after the injection (Fig. 7B,C). In other words, the stimulatory effects of osteocyte-derived CCN2 on NFATc1 and CTSK protein levels in BM cells were observed in 2- and 7-month-old mice (Figs 5B,C and 6B,C), but the effect was not observed in 13 months-old mice (Fig. 7B,C). In general, transmission electron micrographs of osteocytes show that young osteocytes, which are located around the bone surface, have a large Golgi apparatus, prominent rough endoplasmic reticulum, and numerous cytoplasmic processes projecting into the surrounding matrix, whereas mature osteocytes, which are located in the deep of bone matrix, are characterized by increased lacunar volume, an electron-dense lacunar surface, condensed nuclei, and numerous cytoplasmic vacuoles^6^. These morphological characteristics suggest that mature osteocytes undergo a decrease in their protein production and release, compared with young osteocytes. Furthermore, it is noteworthy that expression and production of CCN2 are higher in younger cells/tissues than in older cells/tissues^14–16^. Our data of immunofluorescence was also similar to these findings (Figs 6D and S4). Therefore, it is feasible that the release of CCN2 from osteocytes in aged mice would be decreased, resulting in decreased osteoclastogenesis regulated by osteocytes.

It was previously reported that CCN2 is up-regulated by excess mechanical stress in osteocytes and that mechanical stress-produced CCN2 induces osteocyte apoptosis^34^. Based on this finding, we consider that, when mechanical force is applied to mature osteocytes derived from aged mice, CCN2 is up-regulated in the cells, but CCN2 is not secreted due to decreased number of cell processes that contact osteoblasts and osteoclasts on the bone surface. As a result, CCN2 is accumulated into the mature osteocyte, and apoptosis pathways may thereby be activated. In fact, our preliminary data showed that the number of terminal deoxynucleotidyl transferase dUTP nick end labeling (TUNEL)-positive osteocytes was increased in cortical bone tissues of WT mice, compared with that of KO mice 1 year after injection with tamoxifen (Fig. S6). Moreover, it has been discussed that osteocyte apoptosis plays an important role in the maintenance of bone homeostasis^3,34,35^. However, since we used only lively osteocytes in this study, it is unknown whether apoptotic osteocytes have no/less osteoclastogenic activity. It is also unknown why the CTSK protein level in BM cells stimulated by *Ccn2*-deficient osteocytes derived from aged mice was higher than that produced by WT-osteocytes, but some other factors stimulating CTSK production might be released from *Ccn2*-deficient osteocytes in aged mice. So further investigations are needed to reveal the mechanism underlying these phenomena.

Finally, we investigated the effect of CCN2 from osteocytes on osteoblast differentiation. We cultured MLO-Y4 cells embedded into collagen gel including rCCN2 and inoculated MC3T3-E1 cells onto the gel (Fig. 8A). The gene expression levels of osteoblastic differentiation markers in MC3T3-E1 cells were up-regulated by MLO-Y4 cells embedded into collagen gel with rCCN2 (Fig. 8B). These findings suggest that osteocytes stimulated by CCN2 in the gel promoted not only osteoclastogenesis, but also osteoblast differentiation, leading to the total enhancement of bone remodeling (Fig. S7)^36,37^.

In summary, when young osteocytes, which are localized around the bone surface, are subjected to mechanical stresses, they produce and secrete CCN2 via numerous cell projections and promote both neighboring osteoclast formation and osteoblast differentiation; hence bone remodeling is promoted by CCN2 from osteocytes. On the other hand, CCN2 from mature osteocytes, which are located deep in the bone matrix, is also up-regulated by mechanical stresses; and this CCN2 accumulates in the cells due to decreased cell projections, resulting in osteocyte death, probably by apoptosis. As a result, bone remodeling turnover may be controlled (Fig. S7). However, to firmly confirm this hypothesis, further investigation is still needed.

## Methods

### Materials

Alpha-modification of Eagle’s medium (αMEM), fetal bovine serum (FBS), and calf serum (CF) were purchased from MP Biomedicals, LLC (Solon, OH); and fetal bovine serum (FBS) and calf serum (CS) were obtained from Equitech-Bio, Inc. (Kierrville, TX). Plastic dishes and multi-well plates were obtained from Greiner Bio-One (Frickenhausen, Germany). Type I collagen solution (Cellmatrix type I-C and I-A) was purchased from Nitta Gelatin Co. Ltd. (Osaka, Japan). Mirimostim, also known as human macrophage-colony stimulating factor (M-CSF), was obtained from Kyowa Hakko Kirin Co., Ltd. (Tokyo, Japan). Anti-NFATc1 and anti-cathepsin K antibodies were purchased from Santa Cruz Biotechnology (Santa Cruz, CA); anti-CCN2 and anti-glyceraldehyde-3-phosphate dehydrogenase (GAPDH), from Abcam (Cambridge, UK); and anti-β-actin from Sigma (St. Louis, MO). Anti-OPG antibody was from R & D Systems Inc. (Minneapolis, MN). GST-RANKL^13^ and rCCN2^38^ were prepared as previously described. *Ccn2* expression vector was generated as previously described^39^, and *Sox9* expression vector containing 3 × HA tag was kindly provided by Dr. T. Hattori^40,41^.

### *Ccn2*-conditonal knockout mice and isolation of osteocyte-rich fractions

Tamoxifen-inducible *Ccn2* conditional knockout mice (CAG-Cre^Esr1^;*Ccn2*^flox/flox^) were generated by mating *Ccn2*^flox/flox^ mice^20,21^, and CAG-Cre^Esr1^ transgenic mice. The structure of targeting vector for the generation of *Ccn2*^flox/flox^ mice is shown in Fig. S2A. B6. Cg-Tg(CAG-cre/Esr1)5Amc/J mice (CAG-Cre^Esr1^ transgenic mice) were purchased from the Jackson Laboratory (Bar Harbor, ME)^42^. These transgenic mice express Cre recombinase gene under the control of CAG promoter and the recombination occurs in all over the body^42^. In these transgenic mice, the mutant mouse estrogen receptor fused to Cre recombinase does not bind to estrogen at physiological concentrations, but binds to the synthetic ligand, tamoxifen (Sigma), leading to its nuclear translocation. Therefore, to systemically delete *Ccn2* gene from CAG-Cre^Esr1^;*Ccn2*^flox/flox^ mice, we injected these mice (1-month-old; females) intraperitoneally with tamoxifen dissolved in sunflower oil (30 mg/ml) given at a dose of 0.15 mg/g body weight at a frequency of once a day for 3 days. *Ccn2*^flox/flox^ littermates were used as controls. These mice were housed in cages with paper-chip bedding under specific pathogen-free conditions with an inverted 12-h light/12-h dark cycle in a humidity-temperature controlled environment, and were provided standard diet and water *ad libitum*. The animals were maintained in accordance with Fundamental Guidelines for Proper Conduct of Animal Experiment and Related Activities in Academic Research Institutions. An osteocyte-enriched cell population was isolated according to a previous report^25^. Briefly, to isolate the osteocyte-enriched population, 1 month, 6 months or 1 year after the injection with tamoxifen, the mice were euthanized and femurs were isolated from the tamoxifen-treated and control mice. Then, the muscles and periosteum were mechanically removed by using a scalpel. After the bone marrow had been flushed out with serum-free αMEM, the bones were cut into small pieces and treated for 20 min with 0.2% collagenase. The supernatant was aspirated as fraction 1 (Fr1) and the bone pieces were then treated for 20 min with 5 mM EDTA and the solution was aspirated (fraction 2; Fr2). This was repeated and cells released into fraction 5 (Fr5) to 8 (Fr8) were collected; and cells in Fr5 and Fr6 were combined into a single sample, as were those in Fr7 and Fr8, and then these cells were cultured on collagen-coated plates in αMEM supplemented with both 5% FBS and 5% CS for several days. All animal experiment protocols (OKU-2017360, OKU-2017157) were approved by the Animal Care and Use Committee, Okayama University.

### Cell cultures

Mouse osteocytic cell line MLO-Y4 was kindly donated by Dr. Lynda F. Bonewald (Indiana University)^22^, and these cells were inoculated at a density of 1 × 10^4^/cm^2^ and cultured on dishes or multi-well plates coated with Cellmatrix type I-C (Nitta Gelatin; Osaka, Japan) in αMEM supplemented with 5% FBS and 5% CS. The murine macrophage-like cell line RAW264.7 (which can be induced to form osteoclast-like cells) and osteoblastic cell line MC3T3-E1 were routinely cultured in αMEM supplemented with 10% FBS in an incubator at 37 °C with an atmosphere of 5% CO_2_ in humidified air.

### Three-dimensional culture system

A 3-D culture system was constructed by mixing equal volumes of type I collagen solution (Cellmatrix type I-A; Nitta Gelatin) and 2 × αMEM with 5% FBS and 5% CS, adding the desired numbers of MLO-Y4 cells or cells of a mouse osteocyte-rich cell population with or without CCN2, and pouring the mixture into each well of multi-well plates^5^. Then, the solution was allowed to gel at 37 °C for 20 min; and the gel cultures were placed in an incubator with humidified air at 37 °C, 5% CO_2_ in αMEM supplemented with 10% FBS. After incubation for 1–2 days, RAW264.7 or MC3T3-E1 cells were inoculated onto the gel to investigate osteoclastogenesis or osteoblast differentiation, respectively. In some experiments, 3-D cultures were prepared without the RAW264.7 or MC3T3-E1 cells to detect osteocyte functions.

### Western blot analysis

After 3-D cultivation of osteocytes, culture medium was removed and the collagen gels were digested by 1% collagenase at 37 °C for 30 min. Then, the cells pelleted by centrifugation were lysed by cell lysis buffer (20 mM Tris-HCl pH 7.7, 150 mM NaCl, 1 mM EDTA, 1% Triton X-100); and thereafter Western blot analysis was performed as described previously^43^.

### Phalloidin staining

After cultivation, the cells embedded into collagen gel were fixed with 3.7% paraformaldehyde for 1 h at room temperature and made permeable with 0.1% NP-40 in PBS. The cells were then incubated with fluorescein phalloidin (Cytoskeleton, Inc., Denver, Co.), which was prepared according to the manufacturer’s directions^11^. Phalloidin labeling was performed for 1 h at room temperature. After having been washed with PBS 3 times, the cells were observed under a fluorescence microscope.

### Real-time RT-PCR analysis

Total RNA was isolated from MLO-Y4 and osteocyte-rich cell population by using ISOGEN reagent (Nippon Gene, Tokyo, Japan). First-strand cDNA was synthesized with a primerScript^TM^ reverse transcriptase (RT) reagent kit (Takara Shuzo, Tokyo, Japan), and amplification reactions were performed by using a SYBR^®^ Green Real-time PCR Master Mix (Toyobo, Tokyo, Japan) or Luna, universal qPCR master mix (New England Biolabs Inc., Ipswich, MA) and StepOne plus real-time PCR system (Applied Biosystems, Carlsbad, CA), as described previously^11,43^. The nucleotide sequences of the primers and expected sizes of the amplicons are shown in Table S1.

### Osteoclastogenesis

Osteoclastogenesis of RAW264.7 cells or bone marrow (BM) cells on the collagen gel was evaluated based on the production of NFATc1 and cathepsin K, as well as TRAP staining. Briefly, RAW264.7 cells were inoculated at a density of 1 × 10^4^/cm^2^ onto the collagen gel in wells of 12-well plates and incubated in the presence or absence of GST-RANKL at the concentration of 100 ng/ml for 2 days^11^. BMCs were obtained as described previously^13,44^ and cultured in a 5% CO_2_ air at 37 °C in αMEM supplemented with 10% FBS and 50 ng/ml mirimostim (M-CSF) for 3 days^13,44^. Then, the attached cells were collected and inoculated at a density of 7.5 × 10^4^/cm^2^ onto the collagen gel in wells of 24-well plate. Until typical multinucleate cells appeared, the cells were treated with GST-RANKL at the concentration of 100 ng/ml.

### Overexpression of CCN2 or Sox9 by electroporation in MLO-Y4 cells

After MLO-Y4 cells had reached confluence, they were collected with 0.05% trypsin-EDTA. The cells were then re-suspended in Nucleofector solution containing both 2 μg CCN2 expression vector^39^ or Sox9 expression vector containing 3 × HA tag^40,41^ and pmax GFP vector or both negative control vector (empty vector; EV) and pmax GFP vector. Electroporation was performed by using an Amaxa^TM^ mouse dendritic cell Nucleofector^TM^ kit and Amaxa Nucleofector^TM^ II device (Lonza Cologne GmbH, Cologne, Germany) according to the manufacturer’s instructions^11^.

### Indirect immunofluorescence analysis

Femurs of wild-type mice at 7-months and 13-months old were dissected and fixed in 10% formalin overnight at 4 °C. Next, the tissues were decalcified with 10% EDTA for 3 weeks and subsequently embedded in paraffin. Cortical bone sections were prepared and deparaffinized, and treated with hyaluronidase (25 mg/ml) for 30 min at room temperature for epitope retrieval. Then, these sections were made permeable with 0.1% NP-40 in PBS. Indirect immunofluorescence analysis was performed by using an anti-CCN2 antibody as described previously^45^. Finally, the sections were stained with 4’6-diamidino-2-phenylindole (DAPI) to detect the nucleus. Sections stained without primary antibody were used as negative controls, which showed no detectable signals.

### Statistical analysis

Unless otherwise specified, all experiments were repeated at least twice, and similar results were obtained. Statistical analysis for 2 group-comparison was performed by using Student′s *t*-test, and statistical analysis for multi-group comparisons was performed by performing Bonferroni’s test. All data were presented as the mean and standard deviation. All methods were performed in accordance with the guidelines and regulations of *Scientific Reports*.

## Supplementary information

Supplementary information
