# Supplementary material for: Roles of matricellular CCN2 deposited by osteocytes in osteoclastogenesis and osteoblast differentiation
Source: Sci Rep. 2019 Jul 29;9:10913. doi: 10.1038/s41598-019-47285-3 (PMC6662664; doi:10.1038/s41598-019-47285-3)
Supplement: Supplementary file 1 — Supplementary information [file 41598_2019_47285_MOESM1_ESM.pdf]

Roles of matricellular CCN2 deposited by osteocytes in osteoclastogenesis and osteoblast differentiation

Takashi Nishida,[1,2] * Satoshi Kubota,[1, 2] Hideki Yokoi,[3] Masashi Mukoyama,[4] and Masaharu Takigawa[2]

[1]Department of Biochemistry and Molecular Dentistry, Okayama University Graduate School of Medicine, Dentistry and Pharmaceutical Sciences, Okayama, Japan; [2]Advanced Research Center for Oral and Craniofacial Sciences, Okayama University Dental School, Okayama, Japan; [3]Department of Nephrology, Graduate School of Medicine, Kyoto University, Kyoto, Japan; [4]Department of Nephrology, Kumamoto University Graduate School of Medical Science, Kumamoto, Japan.

***Corresponding author:** Takashi Nishida, D.D.S., Ph.D., Department of Biochemistry and Molecular Dentistry, Okayama University Graduate School of Medicine, Dentistry and Pharmaceutical Sciences, 2-5-1 Shikata-cho, Kita-ku, Okayama, 700-8525, Japan.

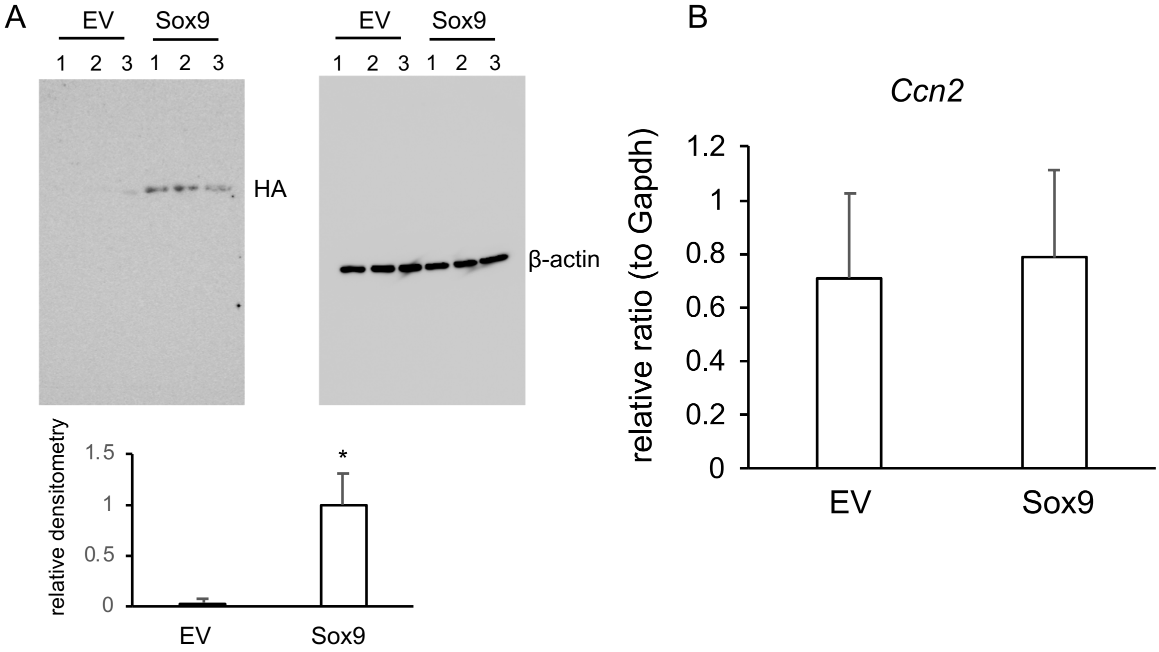

Supplementary Fig. 1. Gene expression of *Ccn2* in MLO-Y4 cells transfected with Sox9 expression vector. (A) MLO-Y4 cells were transfected with a Sox9 expression vector containing 3 x HA tag by using electroporation, and embedded into collagen gel. After the cells had been cultivated for 2 days, cell lysate was collected, and Western blot analysis was performed by using anti-HA antibody. The amount of HA was determined densitometrically and was normalized that of β-actin (n=3). The graph indicates relative density to that with Sox9 expression vector (ratio=1.0) and was analyzed by Student's *t*-test; $p < 0.01$ (*) was considered significant. (B) Total RNA was isolated and real-time PCR analysis was performed by using specific primers for *Ccn2* and *Gapdh*. The amounts of this transcript was normalized to that of *Gapdh* mRNA. Data show the value from independent samples of n=6, and the graph presents the mean and standard deviation.

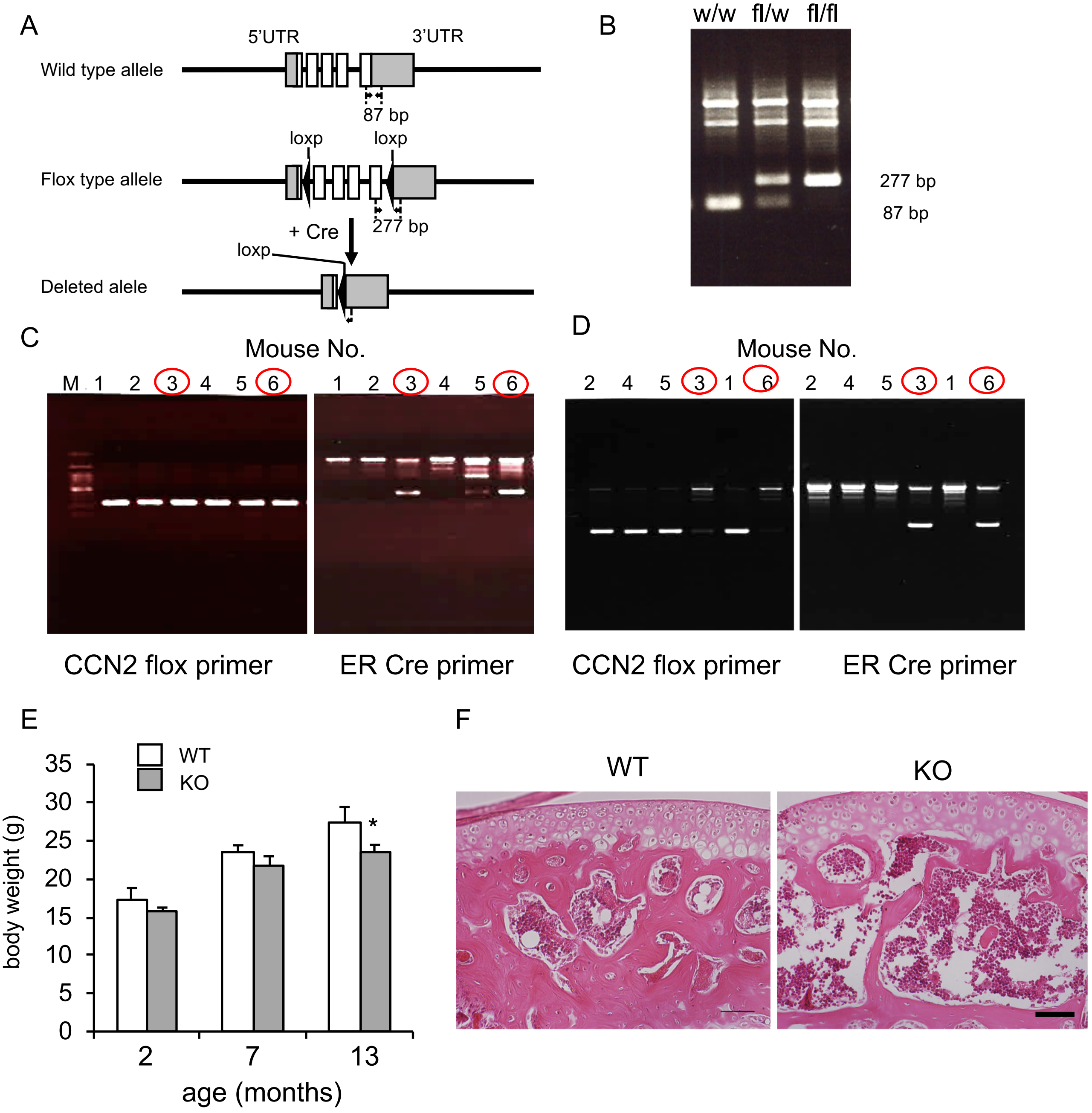

Supplementary Fig. 2. Characterization of CAG-Cre^{Esr1}; *Ccn2*$^{flox/flox}$ mice. (A) Schema of wild type allele and flox type allele. The entire coding region (open boxes; exon 2-5) of the mouse *Ccn2* was flanked by two loxP sites (closed triangles), and recombination product obtained by Cre recombinase activity. Arrows indicate specific primers used in PCR analysis for genotyping. (B) Typical result of genotyping is indicated. PCR products of wild type allele (w/w) and flox allele (fl/fl) are the single bands of 87 bp and 277 bp, respectively. Heterozygous (fl/+) shows two bands of 87 bp and 277 bp. (C) Genomic DNA was extracted from tail of CAG-Cre^{Esr1}; *Ccn2*$^{flox/flox}$ (KO) and *Ccn2*$^{flox/flox}$ (WT) mice at 1 month-old before the treatment with tamoxifen. These mice are littermates, and PCR analysis was performed by using *Ccn2* flox primers and ER Cre primers. Red circles indicate KO mice (mouse No. 3 and 6). (D) Genomic DNA was extracted from tail of WT and KO mice 1 month after the injection with tamoxifen at a dose of 0.15 mg/g body weight for 3 days. *Ccn2* genomic excision was assessed by using PCR (mouse No. 3 and 6; red circles). (E) The graph indicates the body weight of WT and KO mice 1 month, 6 month and 1 year after the injection with tamoxifen. The body weight of KO mice at 13 month-old is significantly reduced, compared with that of WT mice (asterisk $p < 0.05$). (F) Histological examination of the secondary ossification centers of tibiae from WT and KO mice. These sections were stained with hematoxylin-eosin(H-E), and the bone mass of KO tibiae tended to be decreased, compared with that of WT mice. The bar represents 150 μm.

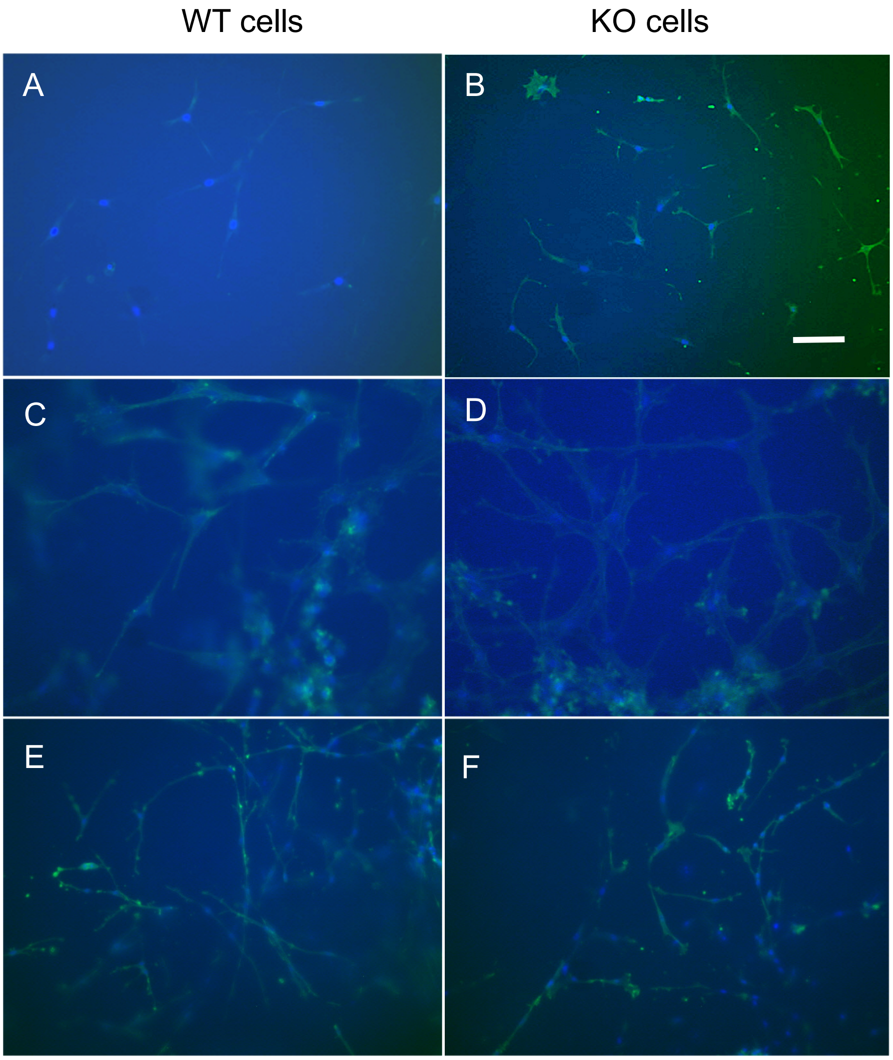

Supplementary Fig. 3. Fluorescent phalloidin staining of osteocyte-enriched populations embedded into collagen gel from WT and KO mice. WT and KO mice at 1 month old were injected with tamoxifen and after 1 month (A and B), 6 months (C and D) and 1 year (E and F), femurs were collected. Then, osteocyte-enriched cells were isolated from WT and KO mice, and these cells were embedded into collagen gel. After 2 days, gel-embedded cells were fixed, and fluorescence staining was performed by using fluorescein phalloidin. The bar represents 100 μm.

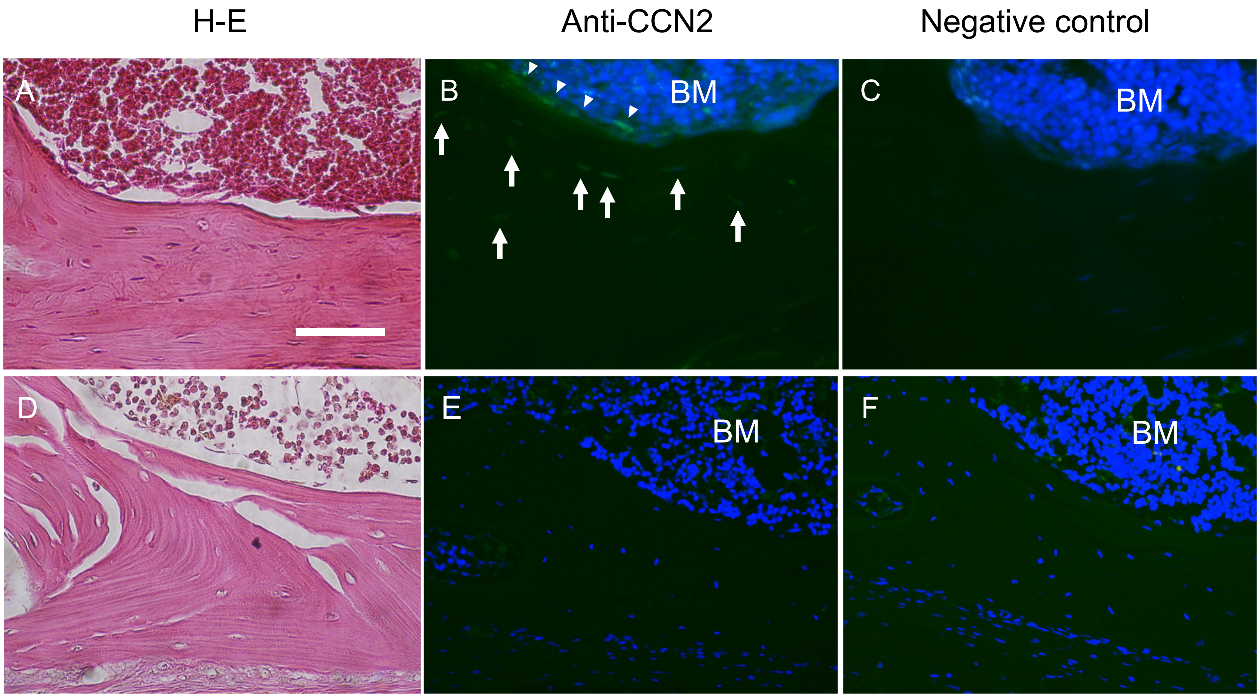

Supplementary Fig. 4. Immunofluorescent localization of CCN2 in cortical bone of WT mice at 7 months-old (A-C) and 13 months-old (D-F). Serial sections were stained with H-E (A, D), or reacted with and without antibody against CCN2, respectively (B, E and C, F). The signals were detected with Alexa-fluor488 conjugated secondary antibody. Immunoreactivity for CCN2 was detected in osteocytes (arrows) and osteoblasts (arrowheads; B), but was not detected in cortical bone from WT mice at 13 months-old (E) . Bar represents 100 μm. Abbreviation “BM” indicates bone marrow.

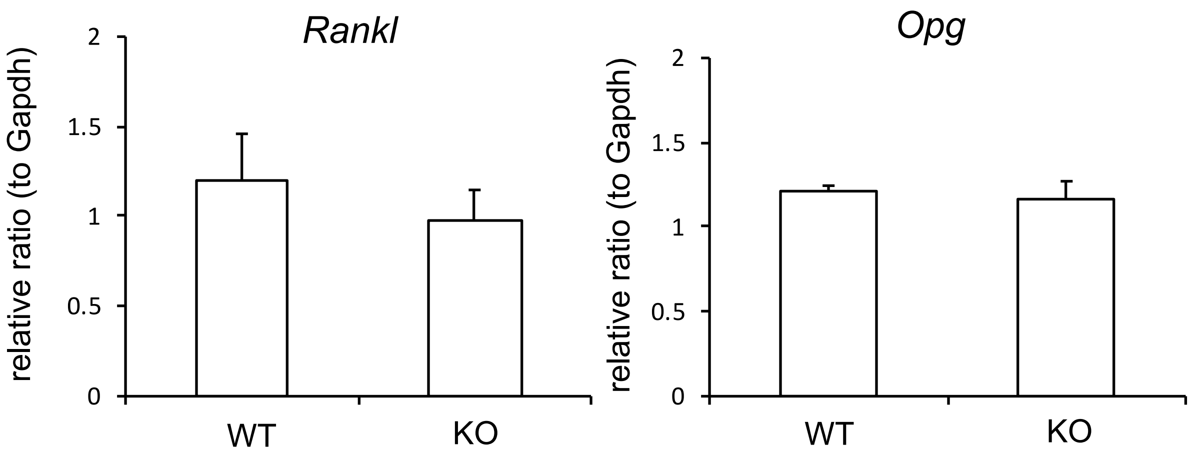

Supplementary Fig. 5. The gene expression of *Rankl* and *Opg* in osteocyte-enriched populations from WT and KO mice. As described in Fig. 4 legend, osteocyte-enriched cell populations were isolated from femurs of WT and KO mice. After 2-D culture for a few days, these cells were embedded into collagen gel and cultured for 5 days. Total RNA was isolated, and real-time RT-PCR analysis was performed by using specific primers for *Rankl* and *Opg*. Data show the value from 3 independent samples, and the graph presents the mean and standard deviation.

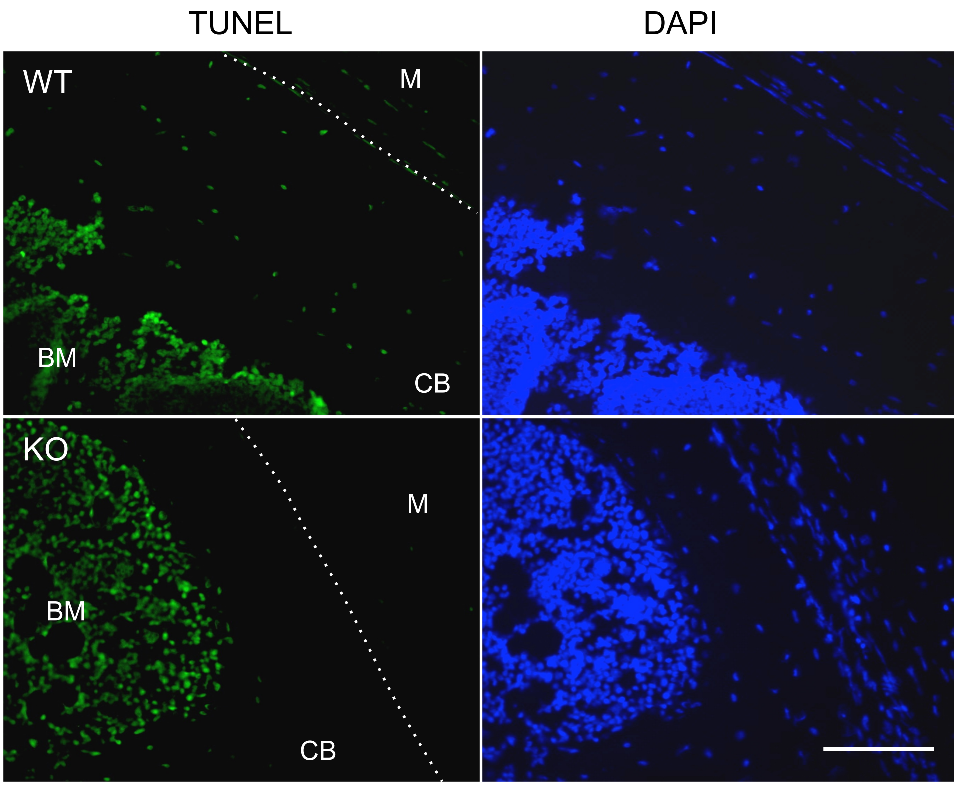

Supplementary Fig. 6. Fluorescent TUNEL staining in cortical bone from WT and KO femurs. The sections of the femur from WT and KO mice 1 year after injection with tamoxifen were prepared, and fluorescent TUNEL staining was performed. The same sections were stained with DAPI. The bar represents 100 µm. Abbreviation “CB”, “BM” and “M” indicate cortical bone, bone marrow and muscle, respectively.

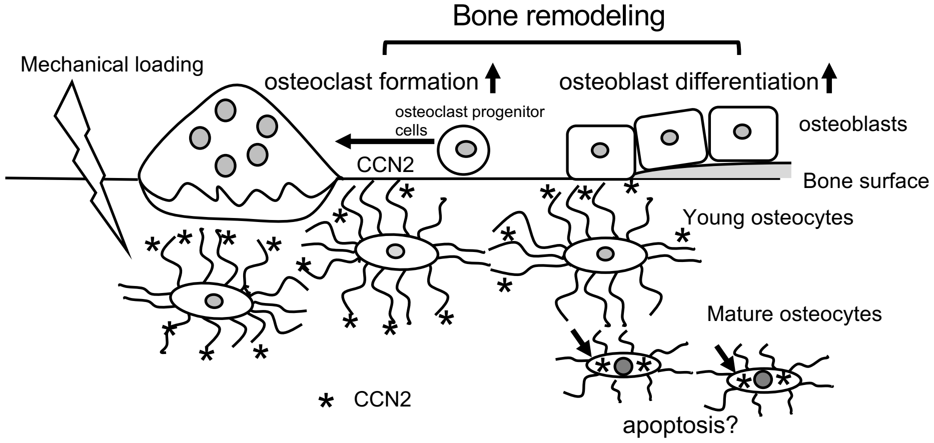

Supplemental Fig. 7. Possible role of osteocytic CCN2 in osteoclastogenesis and osteoblast differentiation. Osteocytes, which are localized around the bone surface, produce CCN2 upon mechanical loading, and this CCN2, which is secreted via numerous cell projections that reach the neighboring osteoclast progenitor cells and osteoblasts, promotes osteoclastogenesis and osteoblast differentiation. On the other hand, mature osteocytes, which are found deep in bone matrix, also produce CCN2 in response to mechanical loading, but this CCN2 accumulates in the cells due to a decrease in the number of cell projections supplying CCN2 to the cells on the bone surface. As a result, apoptosis of osteocytes is induced and bone remodeling may be thus attenuated.

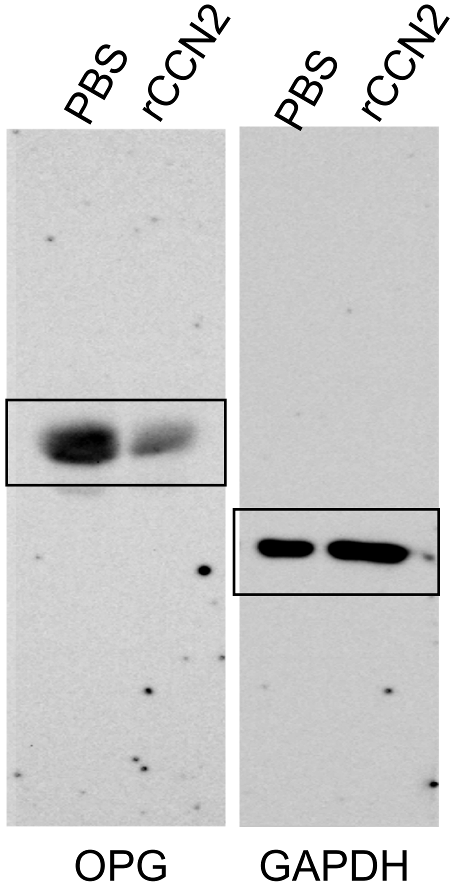

Supplementary Fig. 8. Full-sized blots in Figure 1D. Western blot analysis for OPG and GAPDH. Labels in “PBS” and “rCCN2” represent the cell lysate of MLO-Y4 cells cultured with PBS and rCCN2, respectively.

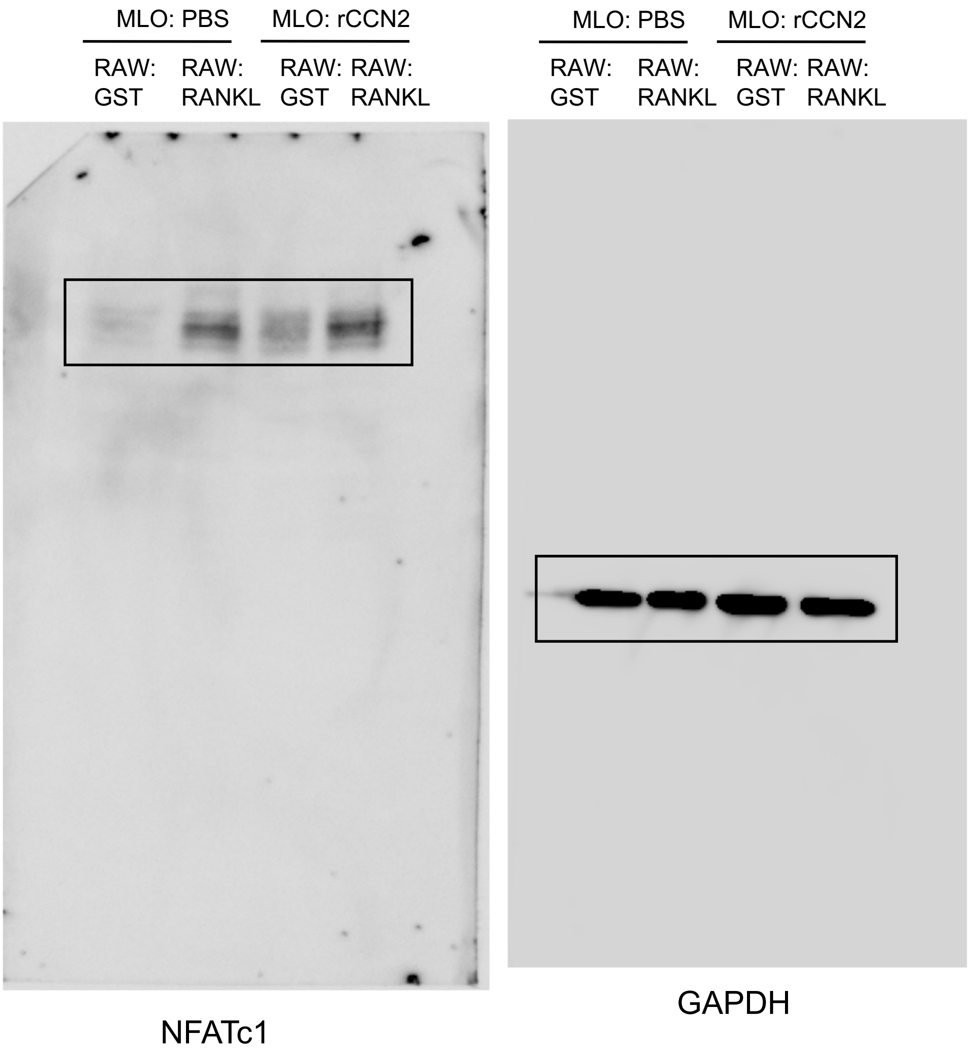

Supplementary Fig. 9. Full-sized blots in Figure 2C. Western blot analysis for NFATc1 and GAPDH. Labels in “MLO; PBS” and “MLO: rCCN2” represent the sample of MLO-Y4 cells embedded in collagen gel with PBS and rCCN2, respectively. Labels in “RAW; GST” and “RAW: RANKL” represent the sample of RAW264.7 cells treated with GST and GST-RANKL, respectively.

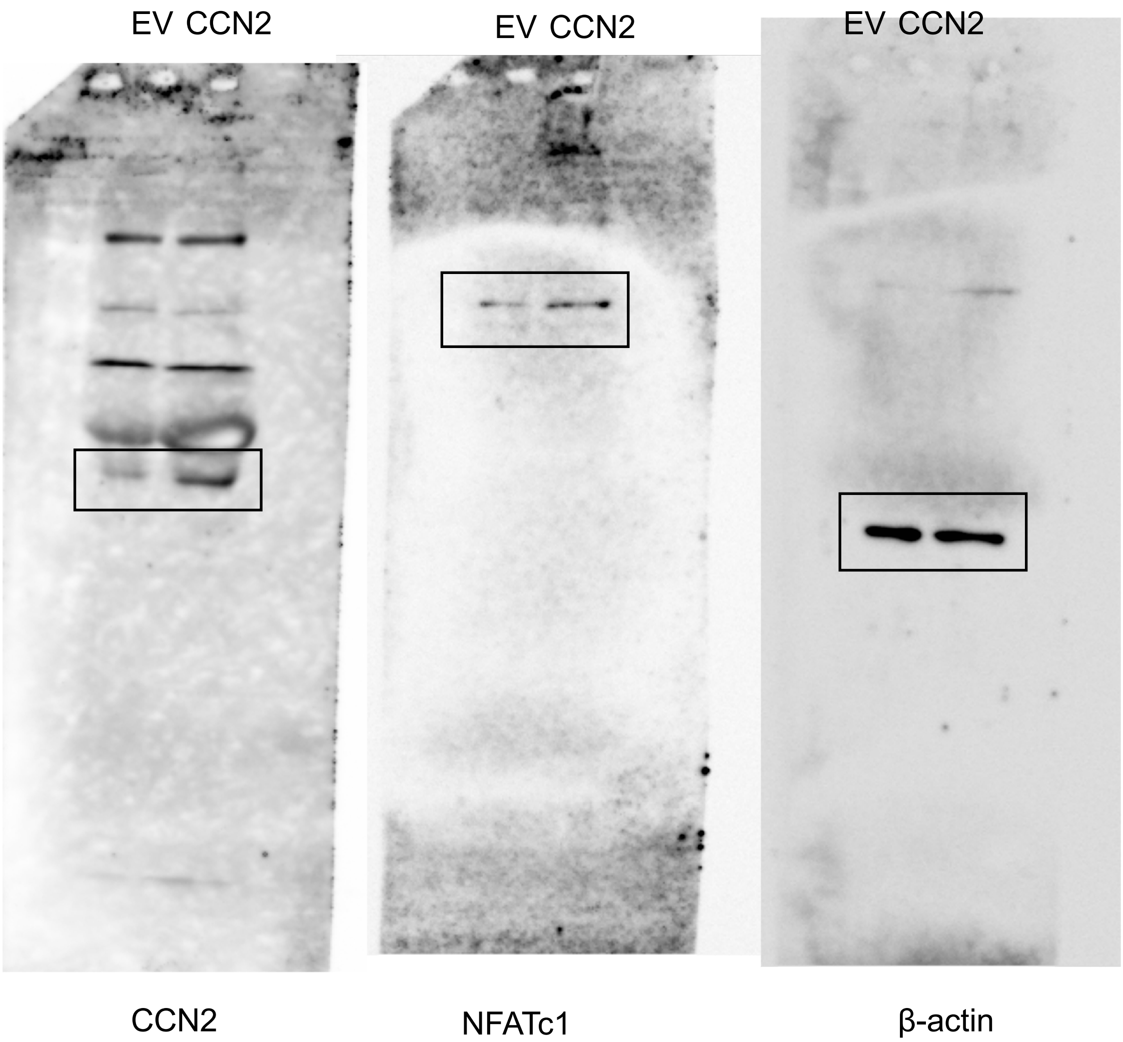

Supplementary Fig. 10. Full-sized blots in Figure 3C. Western blot analysis for CCN2, NFATc1 and β-actin. MLO-Y4 cells were transfected with empty vector (EV), or *Ccn2* expression vector (CCN2). The extra bands detected with anti-CCN2 antibody are non-specific bands,which are thought to originate from serum proteins involved in the 3-dimensional culture system used in this study. In addition, the membrane was re-probed with an anti-β-actin antibody after the stripping of the bound anti-NFATc1 antibody. Therefore, extra bands of a higher molecular weight in β-actin probed membrane are remnant of anti-NFATc1 antibody.

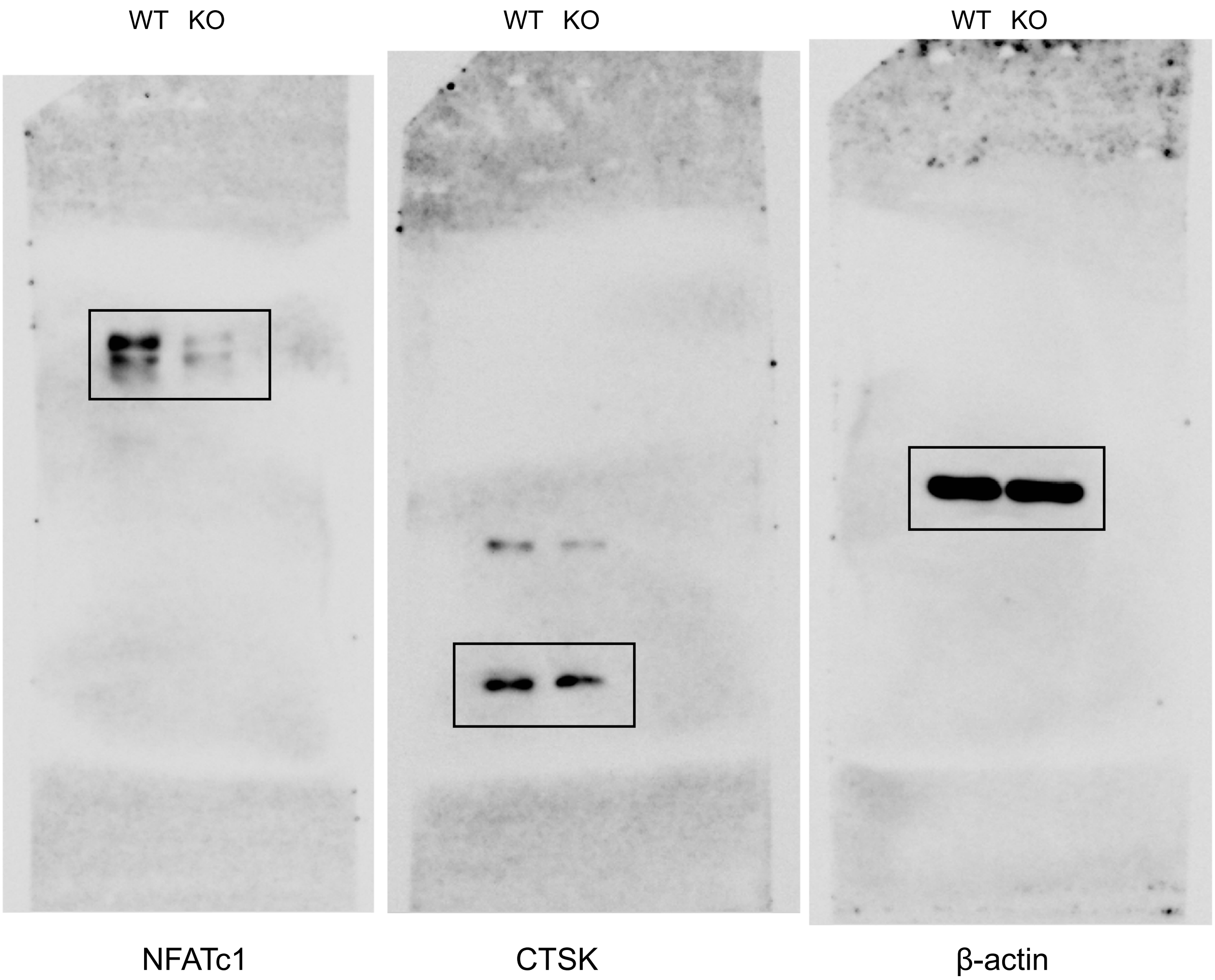

Supplementary Fig. 11. Full-sized blots in Figure 5B. Western blot analysis for NFATc1, CTSK and β-actin. WT; osteocyte-enriched cell population isolated from wild type mice, KO; osteocyte-enriched cell population isolated from CAG-Cre^{Esr1}; *Ccn2*$^{flox/flox}$ mice (*Ccn2* conditional KO mice).

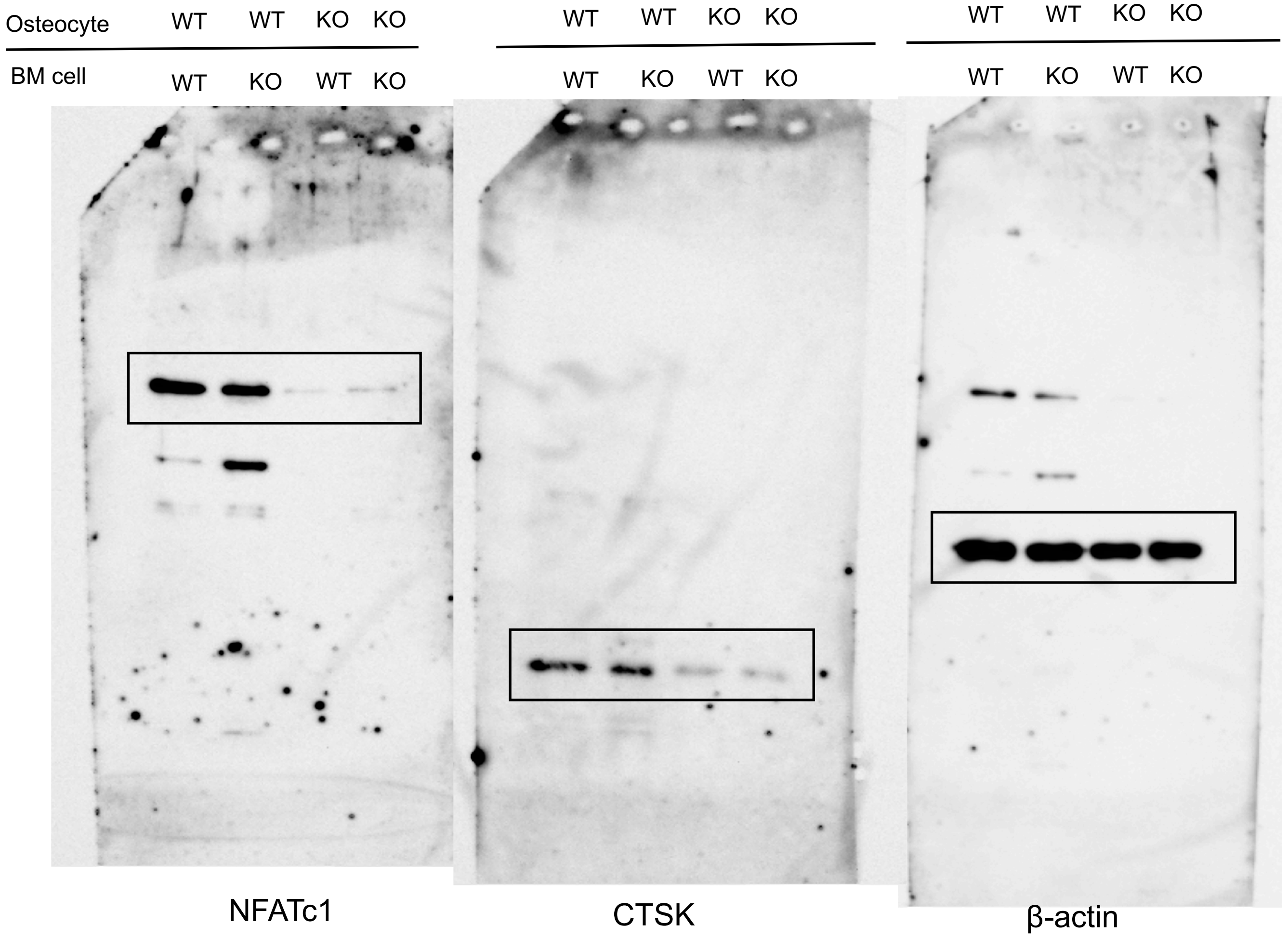

Supplementary Fig. 12. Full-sized blots in Figure 5D. Western blot analysis for NFATc1, CTSK and β-actin in osteocyte-enriched cell population from mice 6 months after the injection with tamoxifen and BM cells (bone marrow cells). WT; wild type, KO; *Ccn2* conditional KO mice. The membrane detecting β-actin was re-probed with anti-β-actin antibody after the stripping of the anti-NFATc1 antibody. Therefore, it is thought that extra bands at higher molecular weight are anti-NFATc1 antibody remnant.

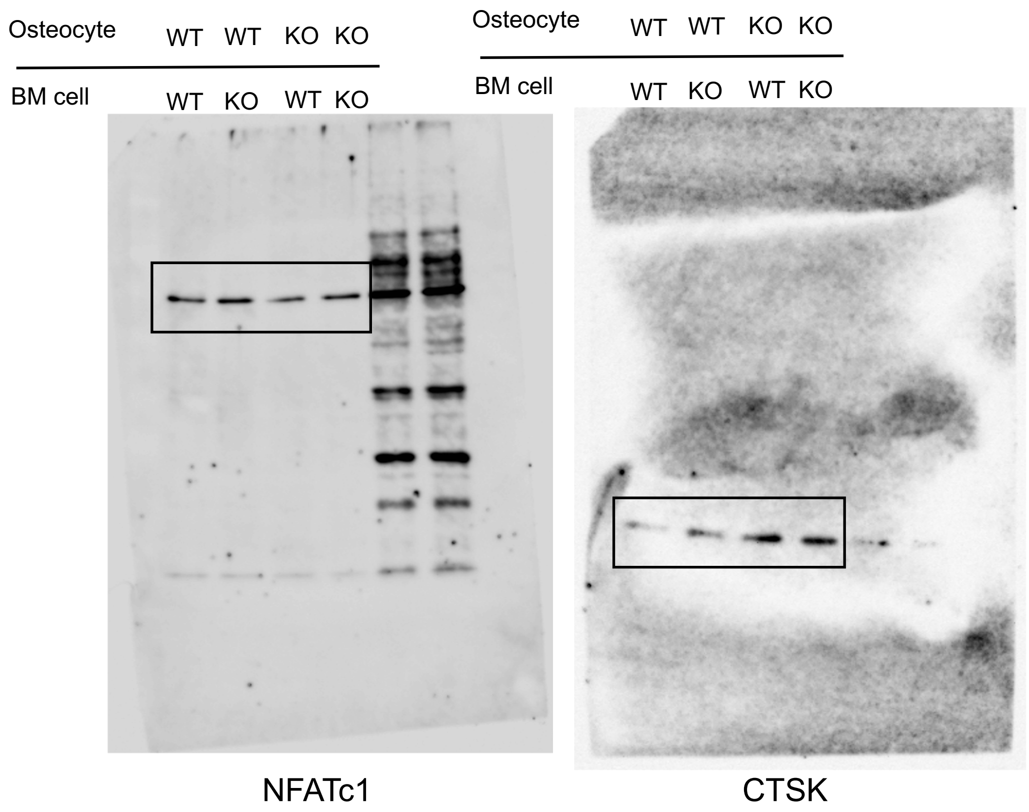

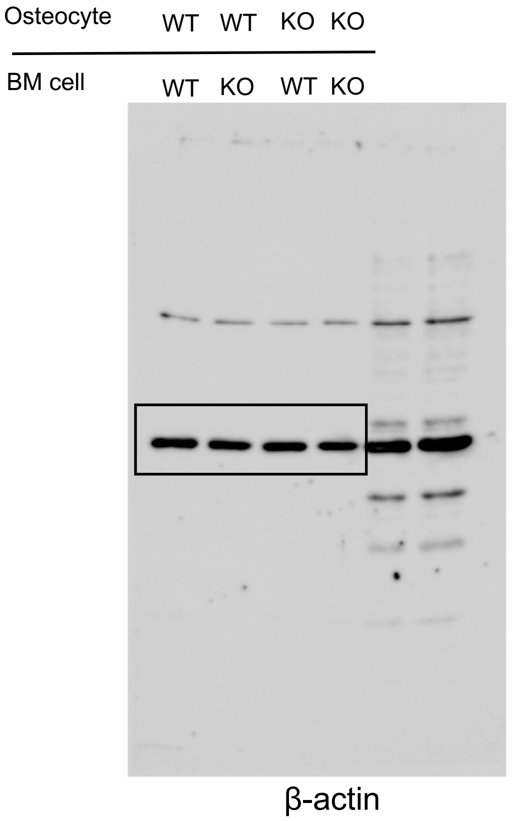

Supplementary Fig.13. Full-sized blots in Figure 6B. Western blot analysis for NFATc1, CTSK and β-actin in osteocyte-enriched cell population from mice 1 year after the injection with tamoxifen and BM cells (bone marrow cells). WT; wild type, KO; *Ccn2* conditional KO mice. The membrane detecting β-actin was re-probed with anti-β-actin antibody after the stripping of the bound anti-NFATc1 antibody and . Therefore, it is thought that extra bands in β-actin probed membrane originate from anti-NFATc1 antibody remnant.

Supplementary Table S1. Mouse forward (F) and reverse (R) primers used for real-time PCR

Gene	Accession No.	Primer sequence	Expected size (bp)
Ccn2	NM_010217.2	(F) 5′-CCACCCGAGTTACCAATGAC-3′<br>(R) 5′-GTGCAGCCAGAAAGCTCA-3′	151
Ocn	NM_001032298.3	(F) 5′- CTCACAGATGCCAAGCCCA- 3′<br>(R) 5′- CCAAGGTAGCGCCGGAGTCT-3′	97
Dmp1	NM_016779.2	(F) 5′-AGATCCCTCTTCGAGAACTTCGCT-3′<br>(R) 5′-TTCTGATGACTCACTGTTCGTGGG-3′	241
Cx43	NM_010288.3	(F) 5′-CTCACCTATGTCTCCTCCT-3′<br>(R) 5′-CTGGCTTGCTTGTTGTAAT-3′	83
Fgf23	NM_022657.4	(F) 5′-GATCCCCACCTCAGTTCTCA-3′<br>(R) 5′-CCGGATAGGCTCTAGCAGTG-3′	193
Rankl	NM_011613.3	(F) 5′-TGGAAGGCTCATGGTTGGAT-3′<br>(R) 5′-CATTGATGGTGAGGTGTGCAA-3′	74
Opg	NM_008764.3	(F) 5′-AGCTGCTGAAGCTGTGGAA-3′<br>(R) 5′-GTTCGAGTGGCCGAGAT-3′	113
Sost	NM_024449.6	(F) 5′-GGAATGATGCCACAGAGGTCAT-3<br>(R) 5′-CCCGGTTCATGGTCTGGTT-3′	80
CTSK	XM_006500974.3	(F) 5′-ATATGTGGGCCAGGATGAAAGTT-3′<br>(R) 5′-TCGTTCCCCACAGGAATCTCT-3′	89
TRAP	NM_007388.2	(F) 5′-CGACCATTGTTAGCCACATACG-3′<br>(R) 5′-TCGTCCTGAAGATACTGCAGGTT-3′	76
Dc-stamp	NM_029422.4	(F) 5′-CTAGCTGGCTGGACTTCATCC-3′<br>(R) 5′-TCATGCTGTCTAGGAGACCTC-3′	305
Alp	XM_006538500.2	(F) 5′-GCTCTCCCTACGCACCCTGTTC-3′<br>(R) 5′-TGCTGGAAGTTGCCTGGACCTC-3′	129
Runx2	NM_001145920.1	(F) 5′-TCGTCAGCATCCTATCAGT-3′<br>(R) 5′-CAGCGTCAACACCATCAT-3′	141
Osx	NM_130458.3	(F) 5′-ACGATGATGATGATGATGATGAT-3′<br>(R) 5′-AACACCAATCTCCTTAACTCTG-3′	81
Gapdh	XM_011241214.1	(F) 5′-GCCAAAAGGGTCATCATCTC-3′<br>(R) 5′-GTCTTCTGGGTGGCAGTGAT-3′	214
